# Association Between B-Cell Marker Expression and *RUNX1* Lesions in Acute Myeloid Leukemia, Beyond *RUNX1*::*RUNX1T1* Fusion: Diagnostic Pitfalls with Mixed-Phenotype Acute Leukemia—B/Myeloid

**DOI:** 10.3390/cancers17081354

**Published:** 2025-04-18

**Authors:** Giby V. George, Malgorzata Kajstura, Audrey N. Jajosky, Hong Fang, Fatima Zahra Jelloul, Andrew G. Evans, W. Richard Burack, John M. Bennett, L. Jeffrey Medeiros, Wei Wang, Siba El Hussein

**Affiliations:** 1 Laboratory Medicine, Department of Pathology, University of Rochester Medical Center, Rochester, NY 14642, USA; 2 Department of Hematopathology, The University of Texas MD Anderson Cancer Center, Houston, TX 77030, USA; 3 Department of Pathology, University of Vermont Larner College of Medicine, Burlington, VT 05401, USA

**Keywords:** AML, *RUNX1*, *RUNX1*::*RUNX1T1*, B-cell expression, flow cytometry analysis, AML-myelodysplasia-related, plasmacytoid dendritic cell differentiation, pDC-AML

## Abstract

Acute myeloid leukemia (AML) with *RUNX1*::*RUNX1T1* fusion is well known to often demonstrate aberrant upregulation of CD19 expression. We studied the clinicopathologic and genetic features of 16 cases of AML with various *RUNX1* lesions, including mutations, copy number gains, and translocations other than fusions with *RUNX1T1*. Most of these cases were classified as AML-myelodysplasia-related or AML-post-cytotoxic therapy based on the cytogenetic and molecular work-up. These neoplasms showed partial expression of one or more B-cell antigens by flow cytometry and/or immunohistochemistry, fulfilling the criteria for mixed-phenotype acute leukemia (MPAL)-B/myeloid (i.e., ≥20% blasts expressing B and myeloid lineage antigens) in most cases. These findings suggest that AML cases with *RUNX1* lesions including mutations, copy number gains, and translocations other than *RUNX1T1* fusion, also commonly express B-cell markers, imparting a “mixed-lineage-like” immunophenotype in cases of AML that otherwise fulfill the criteria for other defined subtypes. We present these cases as to caution regarding this potential diagnostic pitfall and favor a diagnosis of AML with *RUNX1* lesion(s) in the setting of a case of AML with myeloid/B-cell antigen expression, a history of myelodysplasia or cytotoxic therapy, the demonstration of pDC differentiation by flow cytometry (generally associated with the presence of a *RUNX1* mutation), and the presence of a *RUNX1* lesion (mutation, copy number gain, and/or translocation exclusive of a rearrangement with *RUNX1T1*).

## Simple Summary

We describe the largest series of acute myeloid leukemia (AML) cases demonstrating varying degrees of B-cell antigen expression associated with various *RUNX1* lesions other than fusion with *RUNX1T1*. These lesions included *RUNX1* mutations, copy number gains, and a rare *RUNX1*::*CBFA2T3* fusion. The majority of our cases were classified as myelodysplasia-related, while the remaining were labeled as post-cytotoxic therapy AML. Our findings demonstrate a striking immunophenotypic resemblance with mixed phenotype acute leukemia (MPAL)-B/myeloid, as most cases fulfilled the criteria for MPAL.

## 1. Introduction

The Runt-related transcription factor 1 (*RUNX1*) gene, formerly referred to as *AML1*, encodes a transcriptional regulator expressed across all hematopoietic lineages [1,2]. Post-translational modifications enable its interaction with the beta subunit of the core binding factor (CBF) complex, thereby promoting or repressing transcription [1,2]. In addition to its oncogenic role in acute myeloid leukemia (AML) associated with t(8;21)(q22;q22)/*RUNX1*::*RUNX1T1* fusion, *RUNX1* aberrations have been implicated in other subtypes of AML and myelodysplastic neoplasms (MDSs) [1].

In contrast to AML with *RUNX1*::*RUNX1T1* fusion, which often presents in younger age patients and is associated with a favorable outcome, *RUNX1* mutations in AML occur in older individuals and are associated with inferior outcomes [3]. Based on these data, the World Health Organization (WHO) introduced a provisional entity in 2016 known as “AML with mutated *RUNX1*” [4]. Presently, however, the 2022 International Consensus Classification (ICC) system categorizes AML with *RUNX1* mutations under AML-myelodysplasia-related (MR), whereas the fifth edition of the World Health Organization (WHO) classification does not make this recognition [1,5]. AML with *RUNX1*::*RUNX1T1* fusion [1,5] and *RUNX1* mutations [6] may notoriously express B-cell marker antigens CD19, CD79a, and PAX5. In our clinical practice, we noted cases of AML with various *RUNX1* lesions (beyond *RUNX1*::*RUNX1T1* fusion and *RUNX1* mutations) with aberrant expression of several B-cell markers, mimicking mixed-phenotype acute leukemia (MPAL)-B/myeloid at diagnosis [7].

In light of these findings, we sought to expand our cohort [7] and report the clinicopathologic and genetic features of 16 cases of AML with various *RUNX1* lesions, including mutations, copy number gains, and translocations, other than fusions with *RUNX1T1*. We found that these cases showed partial expression of one or more B-cell antigens by flow cytometry and/or immunohistochemistry, raising concern for MPAL-B/myeloid at diagnosis. Given that the prognosis for MPAL-B/myeloid is known to be inferior to that of AML and that the outcomes for AML with various *RUNX1* lesions are still under investigation, we present these 16 cases of AML with various *RUNX1* lesions to bring attention to their unusual immunophenotypic presentation. To our knowledge, this finding has not been widely reported in the literature and deserves awareness, as it may present a potential diagnostic pitfall.

## 2. Materials and Methods

### 2.1. Case Selection

We queried the University of Rochester Medical Center (URMC) molecular diagnostics database and cytogenetics records from 2018 to 2024 for cases of AML involving the bone marrow with various *RUNX1* lesions (mutations, amplifications, and translocations exclusive of a fusion with *RUNX1T1*) and identified 16 cases. These cases were observed to show the expression of one or more B-cell antigens by flow cytometry and/or immunohistochemistry (IHC).

Peripheral smears, bone marrow aspirates, touch imprints, and core biopsy specimens were reviewed in all available cases. The diagnosis of AML-myelodysplasia-related (AML-MR), or AML-post cytotoxic therapy was based on the criteria specified in the WHO classification and the International Consensus Classification (ICC) [1,5]. Relevant clinical information was obtained by reviewing electronic medical records. This study was approved by the URMC institutional review board.

### 2.2. Flow Cytometry and Immunohistochemistry

Cell surface markers were evaluated by flow cytometry (Navios, Beckman Coulter, Indianapolis, IN, USA) using the 10-color myeloid panels (Beckman Coulter) and analyzed using the Kaluza C analysis software version 1.2 (Beckman Coulter Inc., Brea, CA, USA).

Following decalcification using EDTA, 4–6 µm-thick formalin-fixed, paraffin-embedded (FFPE) bone marrow core biopsy sections were prepared. Sections were stained with hematoxylin and eosin. Immunohistochemistry for PAX5 was performed on available bone marrow biopsy core or clot sections (total of nine cases) from 2022 to 2024. The Agilent Dako Omnis automated immunostainer (Agilent, Santa Clara, CA, USA) was used. Heat-induced antigen retrieval was performed on deparaffinized FFPE tissue sections. All available bone marrow cores were incubated with an antibody specific for PAX5 (DAK-PAX5 clone, Agilent Dako, Santa Clara, CA, USA).

### 2.3. Chromosomal Giemsa Banding and Fluorescence In Situ Hybridization

A total of 20 metaphase spreads were analyzed by Giemsa (G) banding for chromosomal analysis. Fluorescence in situ hybridization (FISH) was performed on 200 interphase nuclei. The following AML panel probes (Abbott Molecular/Vysis, Inc., Des Plaines, IL, USA) were used: LSI EGR1 (5q31) SO/D5S23, D5S721 SG; LSI D7S486 (7q31) SO/CEP 7 (D7Z1) SG; LSI RUNX1T1/RUNX1 Dual Color, Dual Fusion Translocation; LSI MLL Dual Color, Break Apart Rearrangement; LSI PML/RARA Dual Color, Dual Fusion Translocation; LSI CBFB Dual Color, Break Apart Rearrangement; LSI 13 (13q14) SG/LSI TP53 (17p13.1) SO; and LSI RARA Dual Color, Break Apart Rearrangement probes.

### 2.4. Next-Generation Sequencing

Targeted DNA-based next-generation sequencing (NGS) was performed on peripheral blood or bone marrow using the 34-gene Illumina Truseq Myeloid Panel on the Illumina MiSeq NGS platform (Illumina Inc., San Diego, CA, USA). The genes included in the panel are as follows: *ASXL1*, *CSF3R*, *EZH2*, *IDH1*, *KIT*, *MYD88*, *NRAS*, *RUNX1*, *SRSF2*, *TP53*, *ZRSR2*, *BCOR*, *DNMT3A*, *FLT3*, *IDH2*, *KRAS*, *NOTCH1*, *PHF6*, *SETBP1*, *STAG2*, *U2AF1*, *BRAF*, *ETV6*, *GATA2*, *JAK2*, *MPL*, *NPM1*, *PTPN11*, *SF3B1*, *TET2*, and *WT1*.

## 3. Results

### 3.1. Clinical Findings

The study cohort included seven women and nine men with a median age of 69.5 years at diagnosis (range: 45–91 years). Eleven patients died either shortly following diagnosis or during treatment. The clinical features of the patients in the cohort, including their prior malignancies (if any) and relevant treatments, are summarized in Table 1.

Three patients were found to have developed AML secondary to cytotoxic therapy for malignancies. Case C had a history of localized prostate cancer treated with prostatectomy, and esophageal adenocarcinoma treated with neoadjuvant chemoradiotherapy (CROSS regimen consisting of radiotherapy followed by five cycles of paclitaxel/carboplatin) and esophagectomy. Five years later, he developed AML and died shortly after diagnosis. Case D had a history of ovarian cancer for which she received several chemotherapeutic agents including carboplatin, paclitaxel, cisplatin, and olaparib. Five years later, she was diagnosed with AML and received five cycles of decitabine and venetoclax. She continued to progress and was switched to supportive management after which she succumbed to disease. Case M was initially diagnosed with stage IA nodular sclerosis Hodgkin lymphoma of the right axilla treated with two cycles of vincristine, doxorubicin, and dexamethasone followed by involved-field radiation therapy. Two years later, he was diagnosed with stage IA adenocarcinoma of the lung (right upper lobe), which was treated with stereotactic radiosurgery. Nine years later, he developed a therapy-related myelodysplastic neoplasm, for which he received 12 cycles of decitabine and venetoclax. Despite treatment, he progressed to AML and died shortly thereafter.

Thirteen patients were diagnosed with AML-MR according to the criteria set forth by the WHO fifth edition [1]. Of these, seven showed plasmacytoid dendritic cell differentiation by flow cytometry analysis, and five of these cases harbored *RUNX1* mutations as recently described in the literature in cases of AML with plasmacytoid dendritic cell differentiation (pDC-AML) [8,9]. In addition, of these 13 patients with AML-MR, two patients (cases A and F) had a prior history of essential thrombocythemia and received hydroxyurea treatment. Case I had a history of stage IIB prostate adenocarcinoma for which he underwent a transurethral resection of the prostate and received therapy with a gonadotropin-releasing hormone (GnRH) antagonist. Little to no relevant clinical history was available for the remaining patients diagnosed with AML-MR. Case H was initially diagnosed with a myelodysplastic/myeloproliferative-neoplasm not otherwise specified for which she received hydroxyurea for four years prior to developing AML. All treatment regimens and outcomes are summarized in Table 1.

### 3.2. Blast Cytomorphology

**Figure 1:**
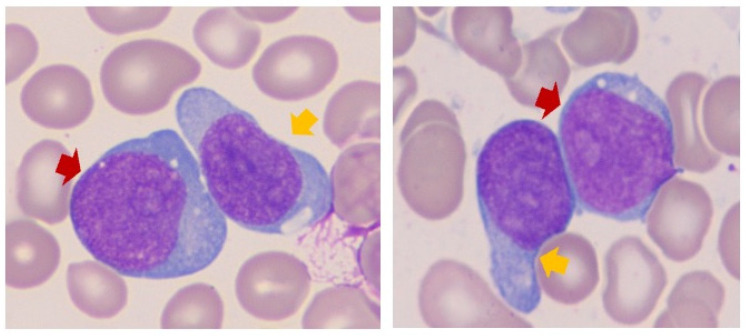
**Illustrative cytomorphology of blasts in a case of AML with a *RUNX1* mutation.** Case A, Wright-Giemsa-stained bone marrow aspirate smear (100× lens objective) demonstrating two populations of immature cells, one being myeloblastic (red arrows) with moderate basophilic cytoplasm, irregular nuclei, finely dispersed chromatin, and prominent nucleoli, while the second population (yellow arrows) shows a cytoplasmic extension reminiscent of plasmacytoid dendritic cell morphology.

The microscopic evaluation of all available peripheral blood and bone marrow aspirate smears, touch imprints, and core biopsy specimens in all cases was consistent with the diagnosis of AML. Only peripheral blood specimens were available for review in three cases (cases G, M, and O). Regardless, in all cases, blasts were intermediate-to-large with moderate-to-intense basophilic cytoplasm and irregular-to-round nuclei containing finely dispersed chromatin, with some nuclei displaying prominent nucleoli (Figure 1).

Plasmacytoid dendritic cell features (cytoplasmic extensions and/or vacuoles) were observed in four cases (cases A, E, I, and J), with all showing pDC differentiation by flow cytometry analysis, as discussed below. Morphologic dysplasia, including granulocytic hypogranulation and hyposegmentation, as well as erythroid nuclear-cytoplasmic desynchrony, was a common finding in cases of AML-MR. Case H showed few blasts with cup-shaped invaginations. A few blasts displayed cytoplasmic azurophilic granules (cases I and K). Cytoplasmic vacuoles were also observed in rare cases (cases K and L), with more striking coalescent vacuoles observed in case M, reminiscent of Burkitt lymphoma cells or vacuolated erythroid progenitors. Finally, cases O and P showed blasts with monocytic differentiation (Supplemental Figure S1).

### 3.3. Immunophenotypic Evaluation

**Figure 2:**
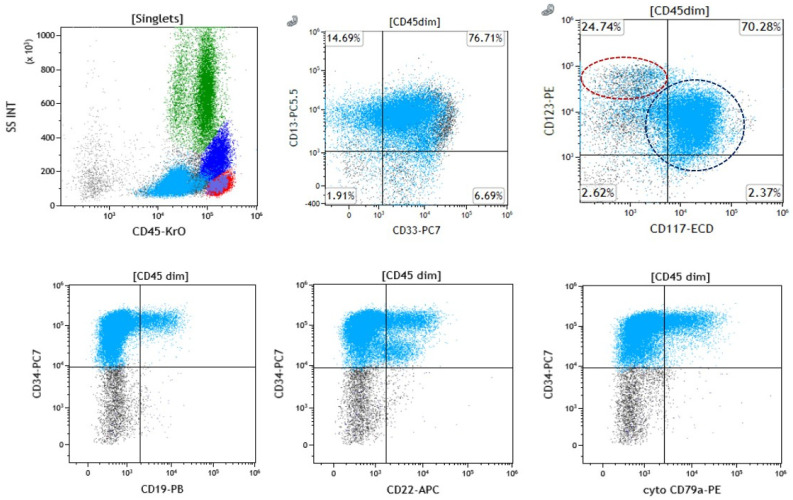
**Illustrative immunophenotypic presentation of a case of AML with a *RUNX1* mutation.** Evaluation by flow cytometry analysis of a marrow specimen from case A shows two populations of CD34-positive immature cells, a minor one with plasmacytoid dendritic cell differentiation with bright CD123 expression (red circle) and a predominant myeloblastic population with dimmer but homogeneous CD123 expression (black circle). Both populations show subset B-cell marker (CD19, CD22, CD79a) expression.

Using flow cytometry, variable CD19, CD20, cytoplasmic CD22, and/or CD79a expression was detected in all available cases (Table 2 and Figure 2).

CD19 was the most frequently expressed B-cell antigen observed in 12 of 16 (75%) cases. CD20 expression (subset) was only observed in 1/16 (6.25%) cases, cytoplasmic (cyto) CD22 expression in 11/16 (68.8%), and surface CD22 expression was observed in 2/16 (12.5%) in lieu of cyto-CD22. Cytoplasmic CD79a expression was observed in 11 of 16 (68.8%) cases (Supplemental Figure S2).

**Figure 3:**
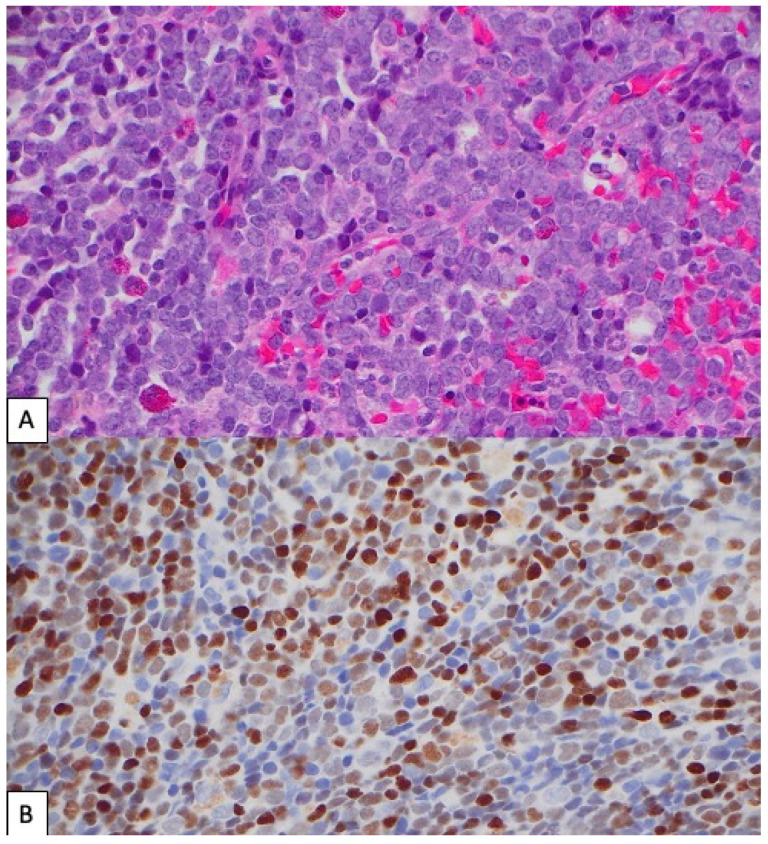
**Illustrative case of AML with a *RUNX1* mutation and PAX5 expression.** (**A**) Hematoxylin and eosin-stained sections of bone marrow trephine biopsy of case I and (**B**) corresponding PAX5 expression in myeloblasts (both 40× lens objective).

The immunohistochemical analysis for PAX5 was performed on nine available specimens (cases A–E, H–J, and O), of which eight were bone marrow core biopsy specimens and one was a bone marrow clot specimen (case I). PAX5 subset positivity was observed in 8 of 9 (88.9%) cases (Figure 3 and Supplemental Figure S3).

The co-expression of all five B-cell markers (CD19, CD20, cyto-CD22, cyto-CD79a, and PAX5) was only observed in case E. Several cases showed the co-expression of CD19, cyto-CD22, cyto-CD79a, and PAX5 (31% [5/16]). Four cases were identified to be B-cell antigen triple expressers (cases D and O: CD19, cyto-CD79a, and PAX5; case J: CD19, cyto-CD22, and PAX5; case M: CD19, cyto-CD22, and cyto-CD79a). The double-expression of B-cell antigens (CD19 and cyto-CD79a) was observed in one case (case P). Single B-cell antigen expression (solely CD22, either surface or cytoplasmic) was observed in four cases (I, K, L, and N). The remaining immunophenotypic features of each case are described in Table 2. In aggregate, these data show that 14 of 16 (87.5%) cases fulfilled the criteria for MPAL-B/myeloid at diagnosis (i.e., ≥20% blasts expressing B-cell and myeloid lineage antigens) with 12 out of 16 cases (75%) demonstrating the expression of at least two B-cell markers (Table 2).

Eight of 16 (50%) cases (A, B, E, I, J, M, N, and O) showed some degree of pDC differentiation by flow cytometry, characterized by the bright co-expression of CD123 and HLA-DR in a subset of analyzed cells. Of the cases of pDC-AML, we observed cyto-CD22 expression in 87.5% (7/8) of cases, CD19 expression in 75% (6/8), cyto-CD79a expression in 62.5% (5/8), CD20 in 12.5% (1/8), and PAX5 expression by IHC in 83.3% (5/6) of the available cases (Table 2 and Supplemental Figure S2).

MPO expression was observed in nine of 15 (60%) available cases; in the remaining cases, the expression of myeloid lineage-associated antigens, such as CD13, CD33, and/or CD117, was observed. The co-expression of CD34 and HLA-DR was identified in all cases. CD4 and CD7 were the most frequent aberrant T-cell antigens expressed in 9/14 available cases (64.3%) and 10/16 (62.5%) cases, respectively, with co-expression observed in six cases. The myeloid blasts in case J also showed CD10 expression (small subset).

### 3.4. Cytogenetic and Molecular Evaluation

Thirteen of 16 (81.3%) patients were diagnosed with AML-MR according to the molecular and cytogenetic criteria set forth by the fifth edition of the WHO [1]. Mutations in *RUNX1* were observed in 8 of 16 (50%) cases, all in the setting of AML-MR, and five (62.5%) of these cases showed pDC differentiation by flow cytometry analysis. Additional *RUNX1* copy number gains were identified in two patients with *RUNX1* mutations. Copy number gain of *RUNX1* without an associated *RUNX1* mutation was observed in eight (50%) cases. Of these, a rare *RUNX1*::*CBFA2T3* fusion was also observed in one patient (case D). *RUNX1* lesions appeared sub-clonal in at least five cases: three cases with mutations (cases H, I, and J) and two cases with copy number gains (cases L and N).

Among patients diagnosed with AML-MR, other mutations in *SF3B1, NRAS, ASXL1, SRSF2*, *FLT3*, *TET2*, *CBL*, *IDH1/2*, *DNMT3A*, *PHF6*, *PTPN11*, *JAK2*, *EZH2*, *BCOR*, and *TP53*, were also observed. Six cases of AML-MR harbored a complex karyotype. Among the patients diagnosed with AML post-cytotoxic therapy, mutations in *TP53* were seen in all three cases (cases C, D, and M). Monosomy 7 was observed in five cases. Molecular and cytogenetic data for all cases are summarized in Table 3 and Table 4.

## 4. Discussion

In this study, we report the largest series of AML with various *RUNX1* lesions, exclusive of a fusion with *RUNX1T1*, with characteristic B-cell antigen expression shown by flow cytometry and IHC analysis. At diagnosis, the myeloid and B-cell marker expression in the blasts imparted a “mixed immunophenotype-like” presentation, mimicking MPAL-B/myeloid and presenting a diagnostic conundrum. In fact, 87.5% of cases fulfilled the criteria for MPAL-B/myeloid at diagnosis, defined by ≥20% blasts expressing B-cell and myeloid lineage antigens. The bone marrow aspirate of one of the remaining cases (case P) was significantly hemodilute, precluding adequate evaluation, likely leading to underestimation of the total bone marrow blast percentage. Regardless, the detection of MR-genetic aberrations in 13 cases and the history of cytotoxic therapy in three cases superseded the immunophenotypic findings in all 16 cases.

The expression of B-cell antigens, including CD19, CD79a, and PAX5, in AML with t(8;21)(q22;q22) *RUNX1*::*RUNX1T1* is established in the literature [10,11]. Using cell line models, Ray et al. demonstrated the mechanism of PAX5 (or B-cell activator protein [BSAP]) expression in the setting of AML with t(8;21)(q22;q22) *RUNX1*::*RUNX1T1* [10]. In B-cells, the *PAX5* promoter is active enabling transcription. In myeloid precursors, the *PAX5* gene is normally repressed by a polycomb-repressive complex; however, in AML with *RUNX1*::*RUNX1T1*, aberrant mitogen-activated protein kinase (MAPK) signaling, enabled by mutations of *KIT*, *RAS* or *FLT3*, lead to the dissociation of the polycomb repressive complex from the PAX5 promoter, supporting its expression [10]. Accordingly, the promoter of the *CD19* gene is known to be a target of PAX5/BSAP [12]. Other genes known to be under the positive transcriptional regulation of PAX5/BSAP include *N-myc*, *LEF-1*, and *Ig-α* (*mb-1* or *CD79a*) [13,14,15]. The expression of other B-cell antigens, such as CD22, also may be regulated in a similar manner [16].

Previously, Menter et al. reported four cases of AML showing a mixed immunophenotype-like picture at diagnosis (with the expression of CD79a and/or PAX5) [6]. Ancillary testing later revealed point mutations and indels in *RUNX1*, in addition to mutations in *TET2*, *DNMT3A* and *SRSF2*, and tetrasomy 13 in two cases [6]. Our previous [7] and current studies expand on these findings by demonstrating that, in addition to mutations, AML cases with copy number gains, and translocations involving *RUNX1*, other than *RUNX1T1* fusion, may also demonstrate B-cell marker expression, imparting a “mixed-lineage-like” immunophenotype in these cases. Among all the cases included in our study, we found CD19 to be the most frequently expressed B-cell antigen by flow cytometry, observed in 75% of cases. We observed PAX5 subset positivity by IHC staining in 88.9% of cases, when applicable. Case I was notably negative for PAX5, likely due to negativity for the expression of CD19 (as well as CD20 and CD79a). In aggregate, our data showed 12 out of 16 cases (75%) demonstrating the expression of at least two B-cell markers.

The recently described pDC-AML constitutes about 3–5% of all AMLs and has been shown to be characteristically enriched in *RUNX1* mutations, in addition to harboring other clonal hematopoiesis-related mutations [8,9]. pDC-AML is typically composed of two aberrant populations, myeloblasts and pDCs, which are clonally related [14]. pDCs in pDC-AML are characterized by a full spectrum of maturation composed of various stages of pDCs, including early forms expressing CD34 and CD117 with low levels of CD4 and CD303, intermediate forms, and late/mature forms that are completely negative for CD34 and CD117 expression with the high expression of CD4 and CD303 [17,18]. In one series, a subset of cases of pDCs in pDC-AML was shown to exhibit CD22 expression in 60% of cases, with six cases showing uniform expression and 26 cases showing partial expression [9]. Of note, CD22 expression has also been described in the maturation pattern of pDCs [19]. In addition, TDT expression was demonstrated in 37% of the cases [9]. CD19 was negative in all studied cases [9]. In our study, pDCs as delineated by the co-expression of CD123 (bright) and HLA-DR, were particularly enriched in cases of AML-MR (cases A, B, E, I, J, M, N, and O). Of these cases of pDC-AML, we observed cyto-CD22 expression in 87.5% of cases, CD19 expression in 75% of cases, cyto-CD79a expression in 62.5% of cases, CD20 in 12.5% of cases, and PAX5 expression in 83.3% of cases, when applicable. Our findings add to the literature, as the expression of CD20, CD79a, and PAX5 has not previously been reported in pDC-AML. Lastly, we found that cases of pDC-AML were enriched for mutations implicated in clonal hematopoiesis and myelodysplasia, including mutations in genes affecting epigenetic regulation (*TET2* and *DNMT3A*), histone modification (*ASXL1* and *EZH2*), splicing factors (*SRSF2* and *SF3B1*), signal transduction (*NRAS*, *CBL*, and *PTPN11*) and nucleosome assembly (*RUNX1*), refs. [8,9] as well as other variants, distinguishing it from MPAL-B/myeloid. Of note, pDC differentiation was identified solely in cases of AML-MR and was conspicuously absent among cases of AML-MR with *TP53* mutations (all of which were associated with *RUNX1* copy number gain) and AML post-cytotoxic therapy (all of which harbored *TP53* mutations and *RUNX1* copy number gain, with one case demonstrating an additional *RUNX1*::*CBFA2T3* rearrangement), likely reflecting a different pathobiology driven by *TP53* aberrations and impacting the clonal architecture in these cases.

In our cohort, three patients were found to have developed AML secondary to cytotoxic therapy (including alkylating agents and/or anthracyclines) for prior malignancies. Case D had a history of ovarian cancer, for which she also received olaparib. In an analysis of adverse event reports of several poly-ADP ribose polymerase (PARP) inhibitors, Zhao et al. previously reported a strong association between olaparib therapy and the subsequent development of MDS and AML [20]. A rare t(16;21)(q24;q22) *RUNX1*::*CBFA2T3* was observed in this case. *CBFA2T3* (*RUNX11T3* or *MTG16*), a transcriptional corepressor located on chromosome 16q24, is the fifth most common fusion partner of *RUNX1* [21]. Of note, AML with this cytogenetic rearrangement is enriched in therapy-related myeloid neoplasms (77% of cases) [21]. Liu et al. described eight cases of AML with t(16;21)(q24;q22) in addition to 25 cases previously reported in the literature [21]. They noted that prior therapy with a topoisomerase II inhibitor to be the most frequent, followed by exposure to alkylating agents [21]. However, patients with this translocation show similarities to AML with *RUNX1*::*RUNX1T1* with regard to morphology, immunophenotype, gene expression profiling, and response to therapy with a relatively good prognosis (with 70–80% of patients achieving complete remission) [21]. The patient described here continued to progress despite AML treatment with decitabine and venetoclax. Of note, the aberrant expression of CD19 in AML with *RUNX1*::*CBFA2T2* rearrangement has been also recently described [22,23], which supports the theory that various *RUNX1* lesions may impart B-cell marker expression in AML. It is also worth mentioning that although three patients in the cohort were treated with hydroxyurea for a myeloproliferative neoplasm, hydroxyurea therapy has not been associated with an increased risk of developing a secondary malignancy [24]. Although the three cases of AML post-cytotoxic therapy, harboring a *RUNX1* copy number gain (all cases), a complex karyotype (in two cases), and *TP53* mutations (all cases), likely have a different underlying pathobiology, their presentation with a mixed-lineage phenotype is worth mentioning here in order to consider AML with a *RUNX1* lesion, associated with aberrant B-cell expression, in the differential versus the diagnosis of an MPAL-B/myeloid. The mechanism of B-cell expression in cases of AML with *RUNX1* copy number gain has not been elucidated to date, and this association is still largely observational. Future studies will explore the mechanism of B-cell antigen expression in such cases with solely a *RUNX1* copy number gain.

In a study of diagnostic bone marrow and peripheral blood samples obtained from 945 patients with AML (ages 18–60 years), Gaidzik et al. identified 59 *RUNX1* mutations, which predicted chemotherapy resistance, inferior event-free survival, recurrence-free survival, and overall survival [3]. Presently, the ICC classification scheme categorizes AML with mutant *RUNX1* under AML-MR [5], whereas the WHO classification does not make this recognition [1]. Regardless, our case designation of AML-MR was based on the presence of a constellation of genetic lesions, other than *RUNX1*, agreed upon by both the ICC and WHO classifications as myelodysplasia-related (i.e., myelodysplasia-related somatic mutations in cases A, B, E, F, G, and H; complex karyotypes in cases K, L, N, and O; monosomy 7 in case P). Furthermore, the European LeukemiaNet (ELN) classifies *RUNX1* mutations as poor/adverse risk [25]. Our series encompasses a heterogeneous group of patients with different disease pathobiology with diverse genetic intricacies that may be at play (i.e., Myelodysplasia-related and post-cytotoxic therapy), possibly confounding or overriding the potential effects of *RUNX1* lesions [4]. Thus, it is uncertain whether AML with *RUNX1* aberrations conferred inferior outcomes in this particular group of patients that we present. Nevertheless, it is worth describing the challenging immunophenotypic presentations of AML in the setting of myelodysplasia and post-cytotoxic therapy to shed light on the potential B-cell marker expression in such cases. Our study is largely descriptive and observational and limited by its retrospective nature and small number of cases, including few patients with limited clinical notes. Additionally, within our cohort, 11 patients died shortly after diagnosis or during treatment, which limits our understanding of the biologic intricacies in each case. As aforementioned, in the three cases that occurred post-cytotoxic therapy, additional molecular/cytogenetic mechanisms may be at play which contribute to their pathogenesis. It is also important to note that *TP53* mutations are known to independently confer a poor prognosis in AML; hence, it is likely that the presence of this aberration would confound the interpretation of the outcomes of patients with AML with various *RUNX1* lesions. Additionally, *RUNX1* lesions appeared sub-clonal in at least five cases: Three cases with mutations (cases H, I, and J) and two cases with copy number gains (cases L and N). Although we were not able to draw any conclusions regarding the development of the *RUNX1* aberrations, future studies may evaluate the clonal evolution and architecture of these genetic events. Prospective studies with a large cohort and comprehensive molecular genetic data are needed to understand the prognosis in patients with AML with various *RUNX1* lesions, exclusive of *RUNX1T1* fusion and B-cell expression. Sequencing the *PAX5* gene may also aid in the understanding of the underlying molecular mechanisms of this phenomenon. Regardless, the immunophenotypic presentations we describe in this series have not been widely reported in the literature, and we present these cases to increase awareness, as aberrant B-cell expression in such cases may present a potential diagnostic pitfall.

Finally, it is known that AML with t(8;21) (q22;q22) *RUNX1*::*RUNX1T1* fusion generally confers a favorable prognosis in the absence of *KIT* and *FLT3* mutations [26]. On the other hand, MPAL-B/myeloid possesses an outcome intermediate to that of AML and B-cell acute lymphoblastic leukemia (B-ALL) [27]. There is no consensus regarding the treatment regimen for MPAL and few retrospective studies have demonstrated higher remission rates using ALL regimens [27]. Thus, there is potential importance in distinguishing such cases of AML from MPAL-B/myeloid from a therapeutic perspective. The expression of B-cell markers in AML cases may generally be synonymous to MPAL-B/myeloid for oncologists; however, our message in this manuscript expands on scenarios in which AML may show variable degrees of B-cell marker expression but remains a myeloid process rather than a mixed-phenotype process. Knowledge of these nuances, as new molecular and phenotypic features arise in the leukemia research sphere, may enhance the oncologist’s therapeutic decision-making.

## 5. Conclusions

In summary, we describe the largest series of AML cases demonstrating varying degrees of B-cell antigen expression associated with various *RUNX1* lesions other than fusion with *RUNX1T1*. These lesions included *RUNX1* mutations, copy number gains, and a rare *RUNX1*::*CBFA2T3* fusion. Most of our cases were classified as AML-MR, while the remaining were labeled as post-cytotoxic therapy AML. Our findings demonstrate a striking immunophenotypic resemblance between AML with *RUNX1* lesions and MPAL-B/myeloid, as most cases fulfilled the criteria for MPAL-B/myeloid. In general, in the setting of a case of AML with myeloid and B-cell antigen expression, a history of myelodysplasia or cytotoxic therapy, the demonstration of pDC differentiation by flow cytometry, and the presence of a *RUNX1* lesion (mutation, copy number gain, and/or translocation exclusive of a rearrangement with *RUNX1T1*) may favor a diagnosis of AML with a *RUNX1* lesion over a diagnosis of MPAL-B/myeloid. Our findings suggest that various *RUNX1* aberrations may impart an “MPAL-like” phenotype in cases that otherwise fulfill the criteria for distinct subtypes of AML.

## Figures and Tables

**Table 1:** Clinical and pathologic features of cases of AML with *RUNX1* lesions.

Case	Age/Sex	Diagnosis	Prior Malignancy (If Any), Details of Therapy, and Vital Status	Bone Marrow Aspirate or Peripheral Blood Blast Percentage	Fulfilling the Criteria for MPAL-B/Myeloid * by Flow Cytometry Analysis	pDC * Differentiation by Morphology	pDC Differentiation by Flow Cytometry Analysis
**A**	64/F	AML-MR *	JAK-2 positive ET * treated with hydroxyurea; alive	70%	Yes	Yes	Yes
**B**	67/M	AML-MR	No history; alive	80%	Yes	No	Yes
**C**	76/M	AML post-cytotoxic therapy	Localized prostate adenocarcinoma and esophageal adenocarcinoma treated with neoadjuvant chemoradiotherapy (CROSS regimen) and esophagectomy; deceased	60%	Yes	No	No
**D**	59/F	AML post-cytotoxic therapy	Ovarian cancer treated with olaparib; deceased	50%	Yes	No	No
**E**	91/F	AML-MR	No history; deceased	85%	Yes	Yes	Yes
**F**	82/F	AML-MR	ET treated with hydroxyurea; deceased	80%	Yes	No	No
**G**	56/M	AML-MR	No history; alive	85%	Yes	No	No
**H**	70/F	AML-MR	MDS/MPN * treated with hydroxyurea; deceased	80%	Yes	No	No
**I**	77/M	AML-MR	Prostate adenocarcinoma treated with a GnRH * antagonist; deceased	33%	Yes	Yes	Yes
**J**	79/M	AML-MR	No history; alive	16%	No	Yes	Yes
**K**	74/F	AML-MR	No history; deceased	53%	Yes	No	No
**L**	61/M	AML-MR	No history; deceased	30%	Yes	No	No
**M**	70/M	AML post-cytotoxic therapy	Stage IA CHL *, Stage IA NSCLC *, MDS * treated with VAD * (two cycles), FRT *, stereotactic radiosurgery, decitabine/venetoclax (12 cycles); deceased	56%	Yes	No	Yes
**N**	69/M	AML-MR	No history; deceased	68%	Yes	No	Yes
**O**	45/M	AML-MR	No history; deceased	70%	Yes	No	Yes
**P**	61/F	AML-MR	No history; alive	18%	No	No	No

* Abbreviations: AML-MR: Acute myeloid leukemia-myelodysplasia-related; pDC: plasmacytoid dendritic cell; mixed phenotype acute leukemia (MPAL)-B/myeloid (≥20% blasts expressing B-cell and myeloid lineage antigens by flow cytometry). CHL: Classic Hodgkin lymphoma; NSCLC: Non-small cell lung cancer; MDS: Myelodysplastic neoplasm; FRT: Fractionated radiation therapy; ET: Essential thrombocythemia; MDS/MPN: Myelodysplastic neoplasm/myeloproliferative neoplasm; GnRH: Gonadotropin-releasing hormone.

**Table 2:** Immunophenotypic features of AML cases with *RUNX1* lesions.

	Case A	Case B	Case C	Case D	Case E	Case F		Case G	Case H	Case I	Case J
PAX5 *	+ (subset)	+ (subset)	+ (subset)	+ (subset)	+ (subset)	Not performed		Not performed	+ (subset)	−	+ (subset)
**CD2**	−	−	−	−	−	−		−	+ (subset)	−	−
**Surface CD3**	−	−	−	−	−	−		−	−	−	−
**Cytoplasmic CD3**	−	−	−	−	−	−		−	−	−	−
**CD4**	+ (subset)	+ (subset)	−	−	+ (subset)	N/A		+ (subset)	+ (subset)	−	+ (subset)
**CD5**	+ (subset)	−	−	−	−	N/A		−	−	+ (subset)	−
**CD7**	+ (subset)	−	+ (subset)	−	+ (subset)	−		+ (subset)	−	+ (subset)	+
**CD11b**	−	+ (subset)	−	−	−	N/A		+ (subset)	−	+ (subset)	−
**CD13**	+ (subset)	+ (subset)	+	−	+ (subset)	+ (subset)		+ (subset)	+ (subset)	+	+ (subset)
**CD14**	−	−	+ (subset)	−	+ (subset)	−		−	−	−	−
**CD15**	−	−	+ (subset)	+ (subset)	−	+ (subset)		−	−	−	−
**CD19**	+ (subset)	+ (subset)	+ (subset)	+	+ (subset)	+ (subset)		+ (subset)	+ (subset)	−	+ (subset)
**CD20**	−	−	−	−	+ (subset)	−		−	−	−	−
**Cytoplasmic CD22**	+ (subset)	+ (subset)	+ (subset)	−	+ (subset)	N/A**		+ (subset)	+ (subset)	+ (subset)	+ (subset)
**CD33**	+	+ (subset)	+ (subset)	-	+ (subset)	+ (subset)		+ (subset)	+ (subset)	+	+ (subset)
**CD34**	+	+	+	+ (subset)	+	+		+	+	+	+
**CD56**	+ (subset)	−	−	−	−	−		−	−	+	−
**CD64**	−	−	+ (subset)	−	−	−		−	−	−	−
**CD79a**	+ (subset)	+ (subset)	+ (subset)	+ (subset)	+ (subset)	+ (subset)		+ (subset)	+ (subset)	−	−
**CD117**	+ (subset)	−	+ (subset)	+	+ (subset)	+		+ (subset)	+ (subset)	+	+
**CD123**	+ (subset)	+	+	+	+	N/A		+	+	+	+
**HLA-DR**	+	+	+	+	+	+		+	+	+	+
**MPO**	−	−	+ (subset)	+	+ (subset)	−		−	+ (subset)	+ (subset)	-
**TdT**	+ (subset)	+ (subset)	+ (subset)	+ (subset)	+ (subset)	+ (subset)		+ (subset)	+ (subset)	−	+
	**Case K**		**Case L**		**Case M**		**Case N**		**Case O**		**Case P**
PAX5 *	Not performed		Not performed		Not available		Not performed		+ (subset)		Not available
**CD2**	−		−		−		−		−		−
**Surface CD3**	−		−		−		−		−		−
**Cytoplasmic CD3**	N/A		−		−		−		−		−
**CD4**	N/A		+ (subset)		+ (subset)		−		+ (subset)		−
**CD5**	N/A		−		−		−		−		−
**CD7**	+ (subset)		+ (subset)		−		−		+ (subset)		+ (subset)
**CD11b**	N/A		+ (subset)		+ (subset)		−		+ (subset)		−
**CD13**	+		+		-		+		+		+ (subset)
**CD14**	−		-		-		−		−		−
**CD15**	−		+ (subset)		+ (subset)		−		+ (subset)		−
**CD19**	−		−		+ (subset)		−		+ (subset)		+ (subset)
**CD20**	−		−		−		−		−		−
**Cytoplasmic CD22**	N/A **		+ (subset)		+ (subset)		+ (subset)		−		−
**CD33**	+ (subset)		+		+ (subset)		+		+		−
**CD34**	+ (subset)		+ (subset)		+		+		+ (subset)		+
**CD56**	+ (subset)		−		−		+ (subset)		−		−
**CD64**	+ (subset)		+ (subset)		−		−		+ (subset)		-
**CD79a**	N/A		-		+ (subset)		−		+ (subset)		+ (subset)
**CD117**	+		+ (subset)		−		+		+ (subset)		-
**CD123**	N/A		+ (subset)		+		+ (subset)		+		+
**HLA-DR**	+ (subset)		+ (subset)		+		+		+		+
**MPO**	N/A		+ (subset)		+ (subset)		+ (subset)		+		−
**TdT**	N/A		−		+ (subset)		+ (subset)		−		+ (subset)

* PAX5 expression as assessed by immunohistochemical staining; ** Surface CD22 positive (subset); N/A: Not applicable.

**Table 3:** Molecular features of AML with *RUNX1* lesions.

Case	Diagnosis	*RUNX1* Lesion	Pathogenic Mutations (VAF * %)
**A**	AML-MR *	Mutation and copy number gain	*RUNX1* c.318G>T, p.Trp106Cys (68%), *SF3B1* c.2098A>G, p.Lys700Glu (46%), *NRAS* c.34G>A, p.Gly12Ser (42%), *ASXL1* c.2338C>T, p.Gln780Ter (43%)
**B**	AML-MR	Mutations	*RUNX1* c.611G > A, p.Arg204Gln (49%), *RUNX1* c.259_277dup, p.Asp93GlyfsTer51 (28%) *SRSF2* c.284C > G,p.Pro95Arg (47%)
**C**	AML post-cytotoxic therapy	Copy number gain	*TP53* c.856G>A, p.Glu286Lys (87%)
**D**	AML post-cytotoxic therapy	Copy number gain and rearrangement	*TP53* c.273G > A, p.Trp91Ter (26%)
**E**	AML-MR	Mutation	*IDH2* c.419G>A p.Arg140Gln (26%), *SRSF2* c.284C>T p.Pro95Leu (23%), *RUNX1* c.485G>A p.Arg162Lys (25%)
**F**	AML-MR	Mutations	*DNMT3A* c.2645G>A p.Arg882His (40%), PHF6 c.482_483insG p.Ser162LysfsTer10 (8%), *PTPN11* c.211T>C p.Phe71Leu (12%), *RUNX1* c.979delC p.Leu327Ter (18%), *SRSF2* c.284C>A p.Pro95His (43%), *RUNX1* c.508G>A p.Gly170Arg (39%)
**G**	AML-MR	Mutation and copy number gain	*ASXL1* c.2056_2057dupAA, p.Cys687SerfsTer17 (12%), *DNMT3A* c.2645G>A, p.Arg882His (47%), *FLT3* ITD (7%), FLT3 ITD (1%), *IDH2* c.419G>A, p.Arg140Gln (48%), *RUNX1* c.743dupA, p.Asn248LysfsTer13 (89%), *SRSF2* c.284C>T, p.Pro95Leu (50%)
**H**	AML-MR	Mutations	*DNMT3A* c.2645G>A p.Arg882His (48%), *JAK2* c.1849G>T p.Val617Phe (55%) *RUNX1* c.425_426insCCGGC p.Glu143ArgfsTer4 (21%), *TET2* c.4537+1G>A p.? (95%), *RUNX1* c.484A>G p.Arg162Gly (31%)
**I**	AML-MR	Mutation	*ASXL1* c.2077C>T, p.Arg693Ter (31%), *CBL* c.1192C>T, p.His398Tyr (18%), *EZH2* c.1650delG, p.Lys550AsnfsTer125 (71%), *NRAS* c.35G>C, p.Gly12Ala (3%), *NRAS* c.176C>A, p.Ala59Asp (11%), *RUNX1* c.485G>A, p.Arg162Lys (9%)
**J**	AML-MR	Mutations	*BCOR* c.472delA p.Ser158ValfsTer3 (24%), *RUNX1 c*.965C>G p.Ser322Ter (12%), *RUNX1* c.618_619insAACC p.Arg207AsnfsTer7 (4%)
**K**	AML-MR	Copy number gain	*TP53* c.401T>G p.Phe134Cys (84%)
**L**	AML-MR	Copy number gain	*TP53* c.527G>T p.Cys176Phe (61%)
**M**	AML post-cytotoxic therapy	Copy number gain	*PTPN11* c.227A>G p.Glu76Gly (14%), *TP53* c.818G>A p.Arg273His (31%), *TP53* c.752T>A p.Ile251Asn (29%)
**N**	AML-MR	Copy number gain	*TP53* c.376-1_386del p.? (76%)
**O**	AML-MR	Copy number gain	*NRAS* c.182A>G, p.Gln61Arg (47%)
**P**	AML-MR	Copy number gain	*IDH1* c.394C>A, p.Arg132Ser (32%)

* Abbreviations: VAF: Variant allele frequency; AML-MR: AML-myelodysplasia related.

**Table 4:** Cytogenetic features of cases of AML with *RUNX1* lesions.

Case	Diagnosis	*RUNX1* lesion	Karyotype	FISH *
**A**	AML-MR *	Mutation and copy number gain	46,XX,7,+13[15]./48,XX,+13,+21[1]./46,XX[4].	Copy number gains of *RUNX1* (21q22) (5%), monosomy 7 (73%)
**B**	AML-MR	Mutations	46,XY,del(7)(q22)[12]./46,XY[7].	Monosomy 7 (45.5%), *TP53* (17p13.1) deletion (38.5%)
**C**	AML post-cytotoxic therapy	Copy number gain	77<4n>,XXYY,-1,-3,-3,-4,-5,-5,+7,add(7)(q11.2) × 3,-8,+9,-10,-11,-11,-11,-11,-13,-13,-14,-16,add(17)(p11.2)× 2,-19,-20,-21,add(21)(p11.2) × 2, + marx2 [15]./44-45,XY,-5,del(5)(q12q33),-7,add(17)(p11.2)[cp5].	Deletion 5q31 (16.5%), monosomy 7 (4.5%), copy number gains of *RUNX1* (21q22) (70.5%), *RUNX1T1* (8q22) (70.5%), *ABL1* (9q34) (79%), *BCR* (22q11.2) (79%), and *IgH* (14q32) (71%)
**D**	AML post-cytotoxic therapy	Copy number gain and rearrangement	46,XX,der(7)t(7;11)(q22;q13)[19]./46,XX[1]..ish t(16;21)(q24;q22) RUNX1::CBFA2T3 [8/9].	Monosomy 7 (48.5%), copy number gains of *RUNX1* (21q22) (65%) and *MLL* (11q23) (64%)
**E**	AML-MR	Mutation	46, XX	Negative for all tested rearrangements
**F**	AML-MR	Mutations	46,XX[19]., Non-clonal: 46,XX,del(6)(q10)[1].	Negative for all tested rearrangements
**G**	AML-MR	Mutation and copy number gain	47,XY,+13[10]./46,XY[10].	Copy number gains of all probes, suspected genomic doubling
**H**	AML-MR	Mutations	46,XX,+6,inv(6)(p25q13)x2,-20[20]., Non-clonal: add(2)(q32), questionable add(14)(q21)	Copy number loss of *IgH* (14q32) (16%)
**I**	AML-MR	Mutation	46,XY,del(11)(q13q23)[19]./46,XY[1].	Copy number loss of *MLL* (11q23) (84%)
**J**	AML-MR	Mutations	46, XY	Negative for all tested rearrangements
**K**	AML-MR	Copy number gain	54-58<2n>,XX,+X,+1,+2,+4,del(5)(q13q34), +del(5),+6,dic(7;11)(q11.2;q11.2),+9,+10,+11,del(11)(p11.1), +13,+15,-19,+21,+22,+der(?)t(?;13)(?;q14), +der(?)t(?;14)(?;q13),+1-2mar[cp20].	Three *RUNX1* signals (73.5%), three *MLL* signals (72.5%), and three to four PML signals (36.5%)
**L**	AML-MR	Copy number gain	46,XY,del(5)(q22q35),+8,del(17)(p12),-18[1]./46,XY,del(5),del(16)(q21q22),del(17),+mar[1]./ 45-46,Y,del(X)(q21),add(3)(p21),del(5),i(8)(q10),del(9)(q21), der(10)ins(10:?)(q21;?),der(12)t(9;12)(q22;p13),del(16),del(17),-18,+19,-21,i(21)(q10),-22,i(22)(q10),+1-2mar[cp16]./46,XY[2]., Non-clonal: add(6)(q25-27), del(22)(q13)	*EGR1* (5q31) deletion (87.5%), copy number gains of *RUNX1T1* (8q22) (19–49.5%), copy number gains of *RUNX1* (21q22) (19%), and one *CBFB* (16q22) fusion signal (75%)
**M**	AML post-cytotoxic therapy	Copy number gain	66-69,XXY,del(1)(q41),-3,+5,del(5)(q33)x2,- 7,del(7)(q22),+8,der(9;14)(q1 0;q10),+10,+11,add(11)(p11.2),+12,-15,-16,-17,+22,+1-2mar[cp8]./ 46,XY[2]., Non-clonal: +2markers	Hyperdiploidy or near triploidy, three *CRLF2* signals or copy number gains of Xp22.33/Yp11.32 (83.5%), three to four *RUNX1T1* (8q22) signals (85%), three *ABL1* (9q34) signals (89%), four *MLL* (11q23) signals (80%), four *ETV6* (12p13) signals (83.5%), three *IgH* (14q32) signals (80%), three *RUNX1* (21q22) signals (74.5%), four *BCR* (22q11.2) signals (89%)
**N**	AML-MR	Copy number gain	46,XY,del(16)(q11.2q23)[2]./45,sl,del(5)(q22q35),add(17)(q23),-20,-21,+22[2]./49-50,sdl1,-7,add(11)(q23),-13,+22,+5-6mar[cp7]./50-51,sdl2,+del(5)[cp3]./50-51,sdl2,+X,+9,+14,-del(16)[cp4]./51-52,sdl4,+8[cp2]., Non-clonal:t(2;8)(p13;p13)	*EGR1* (5q) deletion (80%), D7S486 (7q31) deletion (62.5%), monosomy 7 (9.5%), copy number gains of D5S721/D5S23 (5p15.2) (25%), copy number gains of *RUNX1T1* (8q22) (39.5%), copy number gains of *RUNX1T1* (8q22) and *RUNX1* (21q22) (19.5%), three fused *MLL* (11q23) signals (10%)
**O**	AML-MR	Copy number gain	49,XY,+3,+8,?t(10;21)(q26;q21),+14[13]./46,XY[7]..nuc ish(RUNX1x3)[180/200]..ish ?t(10;21)(3′ or 5′RUNX1+;3′ or 5′RUNX1-)[2/2].	Copy number gains of both *RUNX1T1* (8q22) and *RUNX1* (21q22) (95%)
P	AML-MR	Copy number gain	46,XX,del(7)(q22q35)[4]./(46,idem)x2,-del(7),+8[16].	D7S486 (7q31) deletion (19%), likely tetraploidy in ~50% of cells based on copy number gains of the following: tetrasomy 5 (53.5%), D7Z1 (CEP7) three copies, D7S486 (7q31) two copies (25.5%),loss of chromosome 7 relative to tetraploidy with additional loss of 7q, *RUNX1T1* (8q21) four copies (10%), *RUNX1T1* (8q21) five copies (40%),additional signal for 8q21 relative to tetraploidy, *RUNX1* (21q22) four copies (50%), *KMT2A* (11q23) four copies (47%), *KMT2A* (11q23) three copies (6.5%), loss of 11q relative to tetraploidy, *RB1* (13q14) four copies (49%), *TP53* (17q13) four copies (49%), *PML* (15q22) four copies (46.5%), *RARA* (17q21) four copies, (46.5%), *CBFB* (16q22) four copies (44%), *RARA* (17q21) four copies (50%)

* Abbreviations: FISH: fluorescence in situ hybridization; AML-MR: AML-myelodysplasia related.

## Data Availability

Data is contained within the article or Supplementary Materials.

## Supplementary Materials

The following supporting information can be downloaded at: https://www.mdpi.com/article/10.3390/cancers17081354/s1, Supplemental Figure S1. Cytomorphology of blasts in cases of AML with *RUNX1* lesions. Cases A–Q, Wright-Giemsa-stained bone marrow aspirate and peripheral blood smears (100× lens objective) demonstrate intermediate-to-large-sized blasts with moderate-to-intense basophilic cytoplasm, irregular-to-round nuclei containing finely dispersed chromatin with some displaying prominent nucleoli, and cytoplasmic granules or vacuoles. Plasmacytoid dendritic cell morphology is observed in select cases (cases A, E, F, J, and K). Supplemental Figure S2. Flow cytometry evaluation of cases of AML with *RUNX1* lesions. Cases (A–Q) show the subset expression of B-cell markers CD19, CD20, cytoplasmic CD22, and CD79a. The most frequently expressed B-cell antigen by flow cytometry was CD19, followed by cytoplasmic CD79a and CD22. CD20 expression was only observed in two cases. In addition, nine of 17 (52.9%) cases (A, B, E, F, J, K, N, O, and P) showed some degree of plasmacytoid dendritic cell differentiation, characterized by the bright co-expression of CD123 and HLA-DR in a subset of analyzed cells. Supplemental Figure S3. PAX5 expression in cases of AML with *RUNX1* lesions. Hematoxylin and eosin-stained sections of bone marrow trephine biopsies and a clot specimen of nine cases involved by AML (cases A–E, H–J, and O) and corresponding PAX5 expression in myeloblasts (all 40× lens objective).
