# Supplementary figures and images for: Association Between B-Cell Marker Expression and RUNX1 Lesions in Acute Myeloid Leukemia, Beyond RUNX1::RUNX1T1 Fusion: Diagnostic Pitfalls with Mixed-Phenotype Acute Leukemia—B/Myeloid
Source: Cancers (Basel). 2025 Apr 18;17(8):1354. doi: 10.3390/cancers17081354 (PMC12026294; doi:10.3390/cancers17081354)

Case A

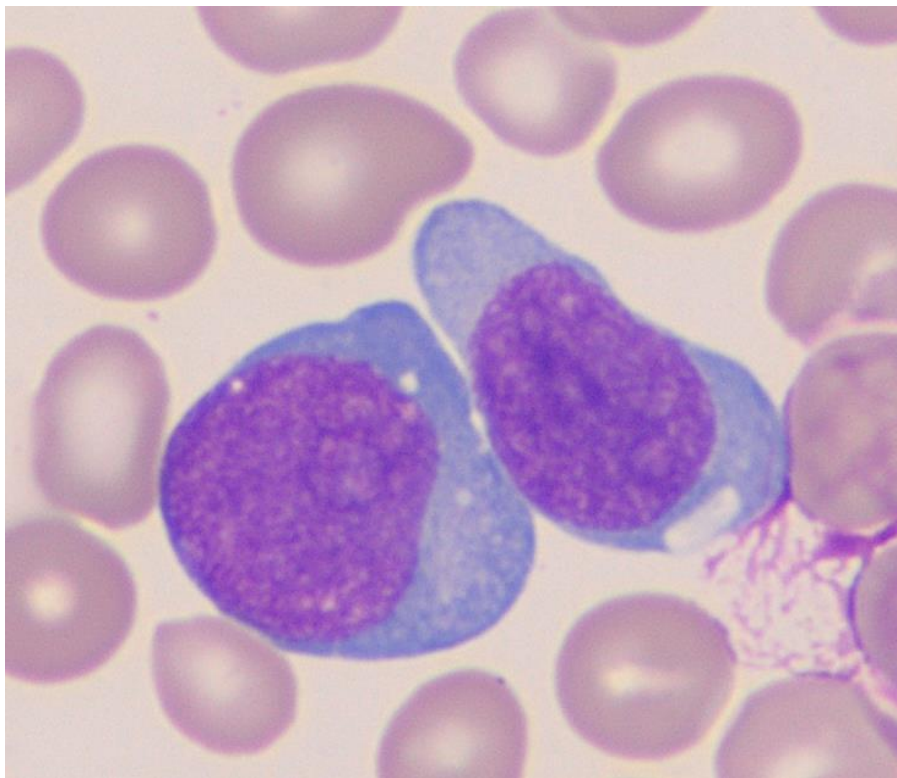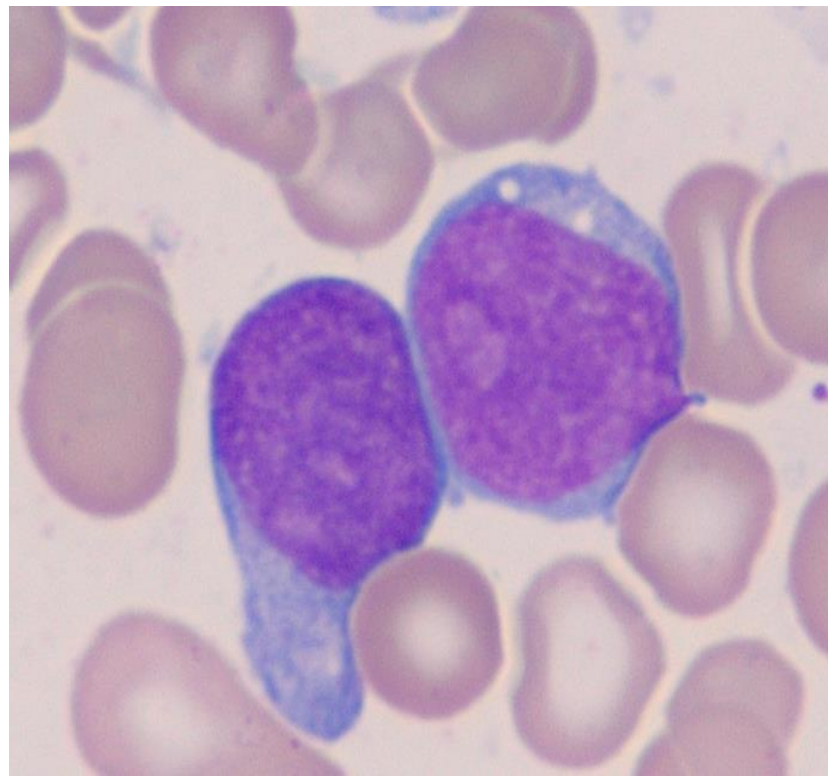

Case B

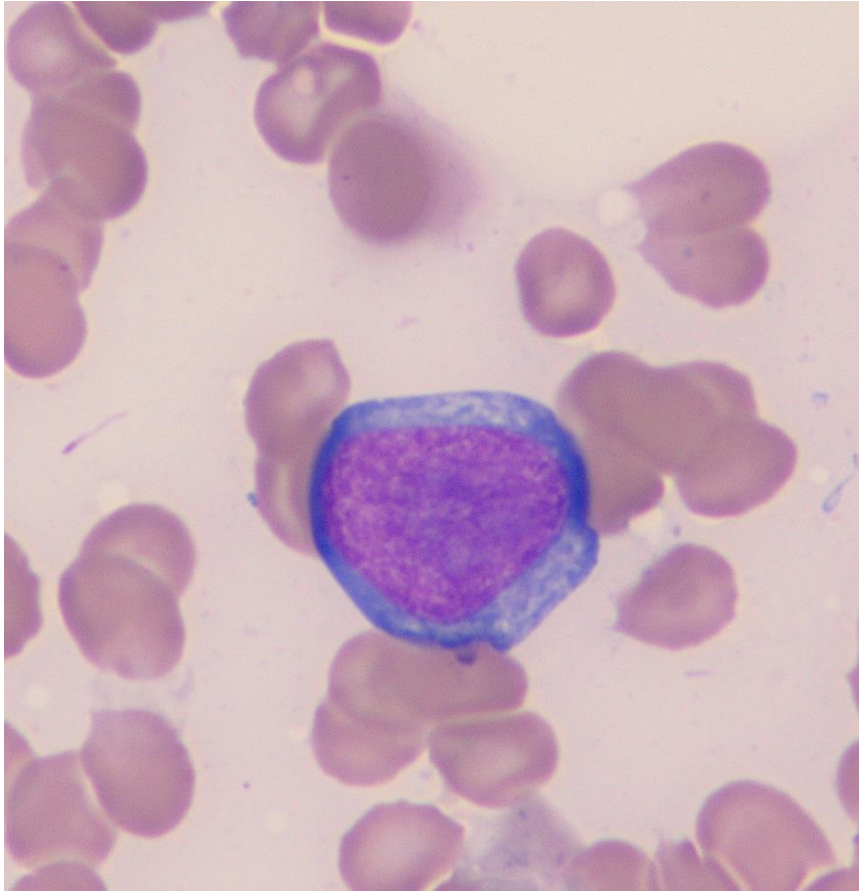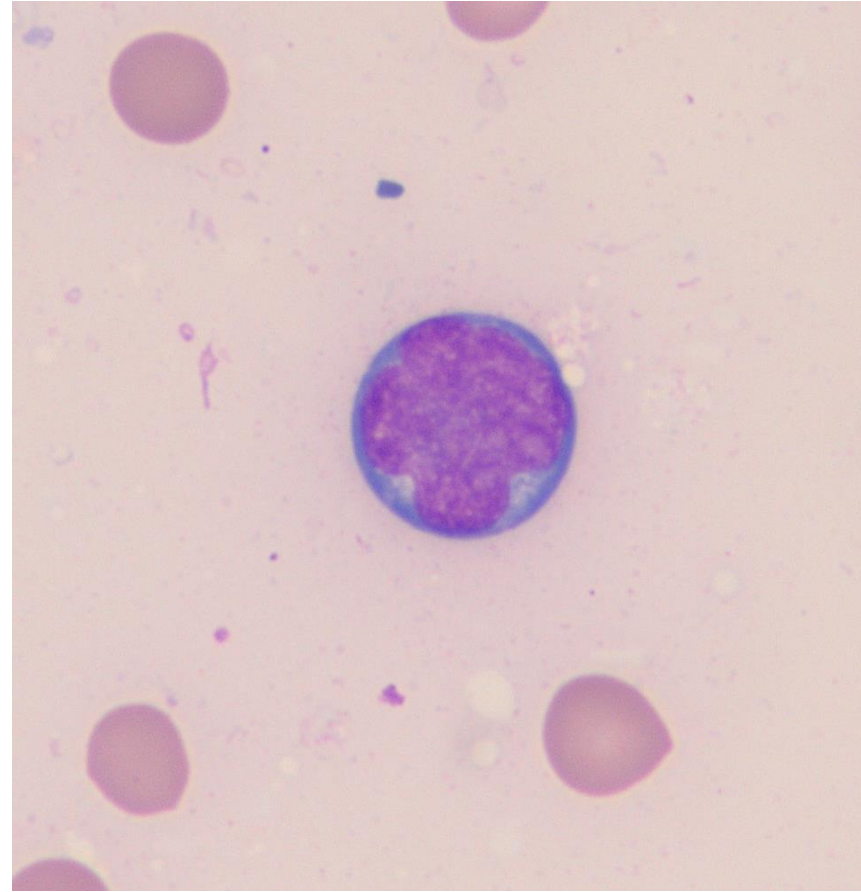

Case C

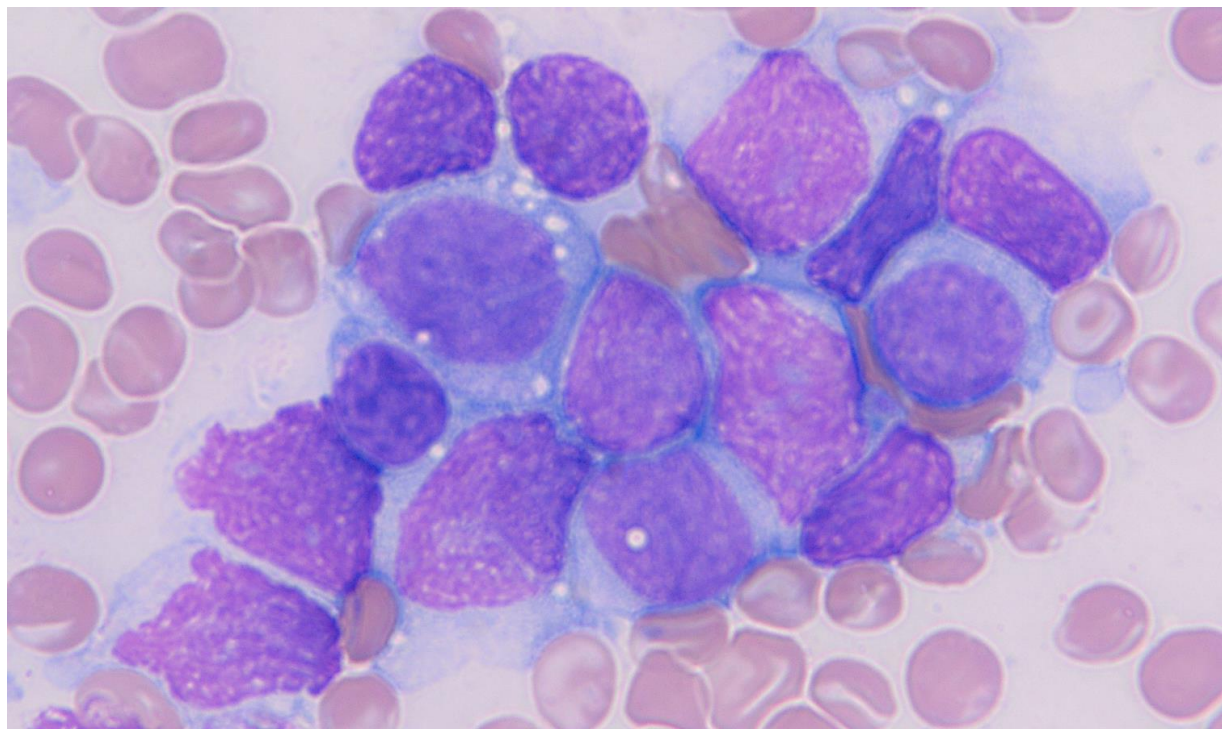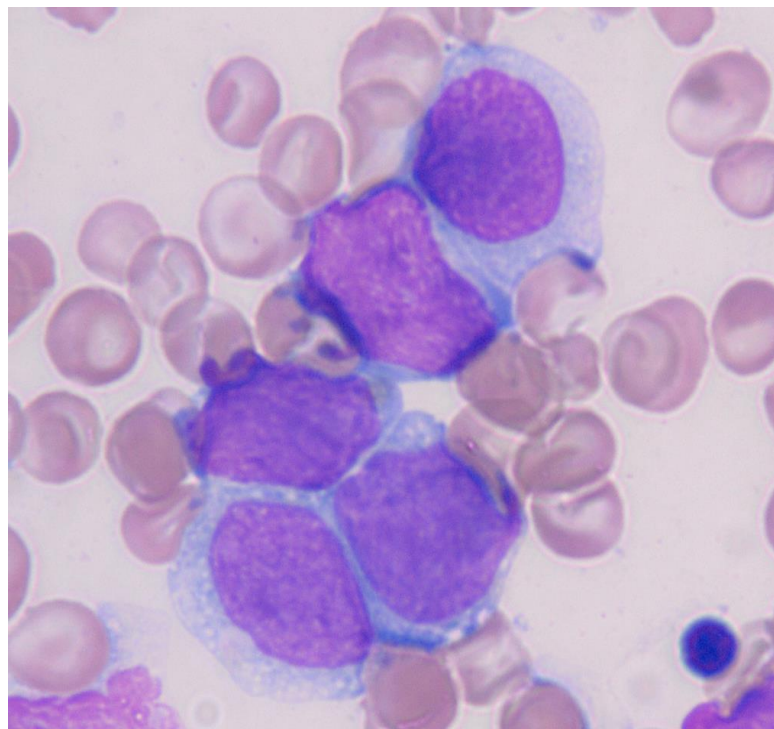

Case D

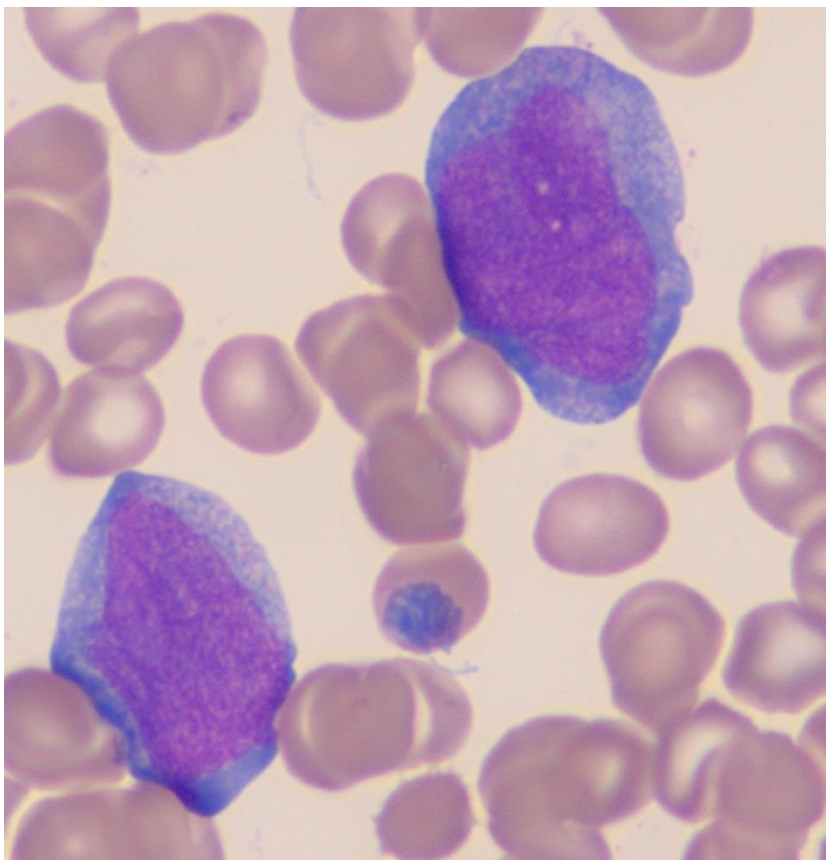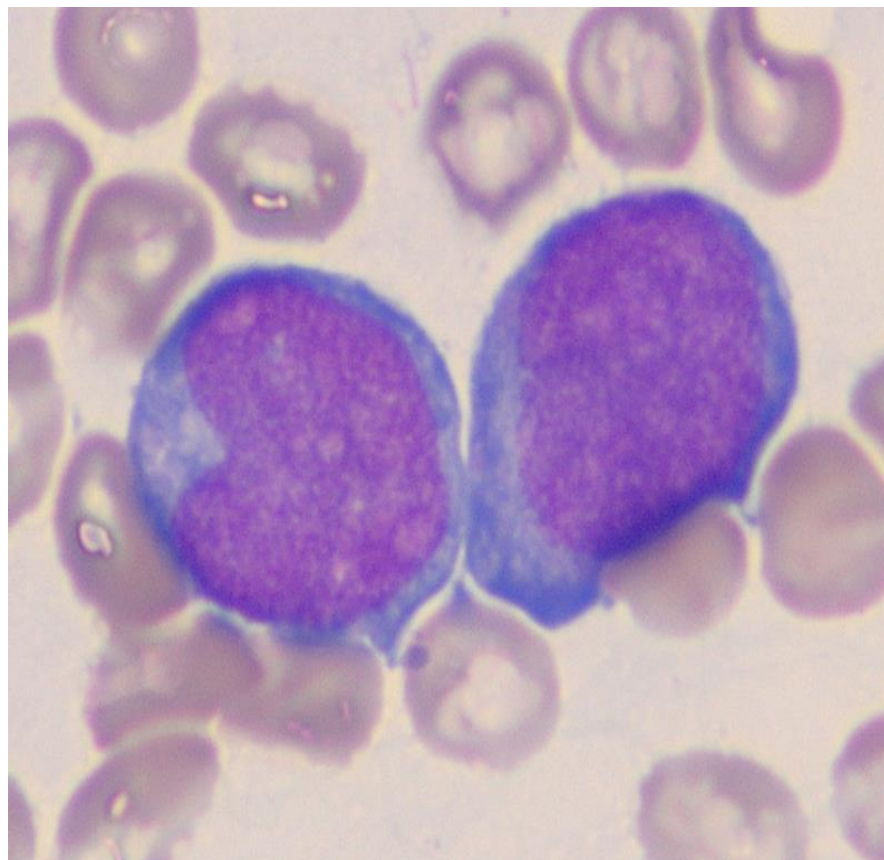

## Case E

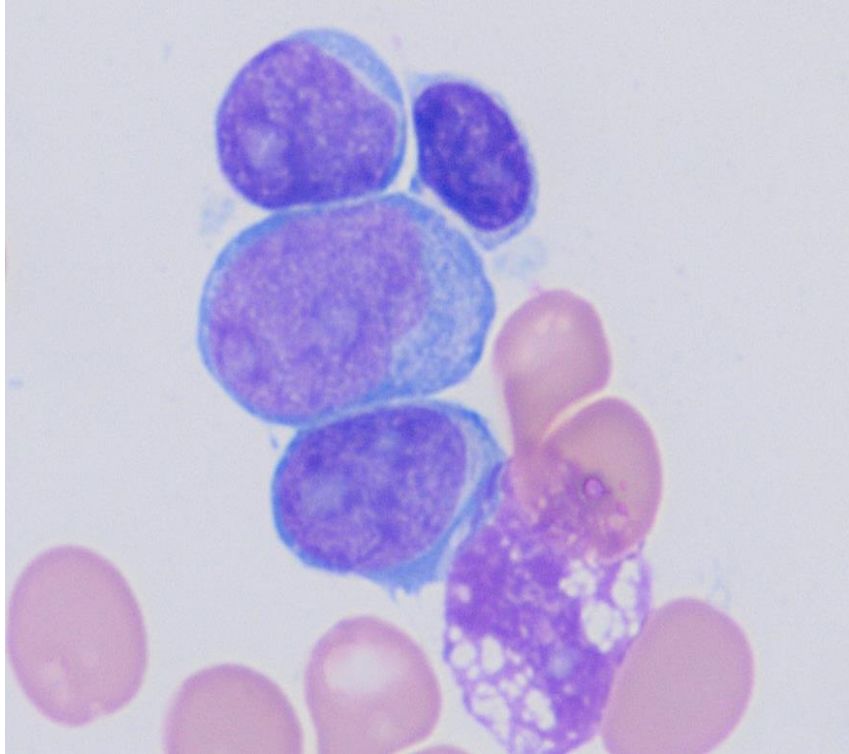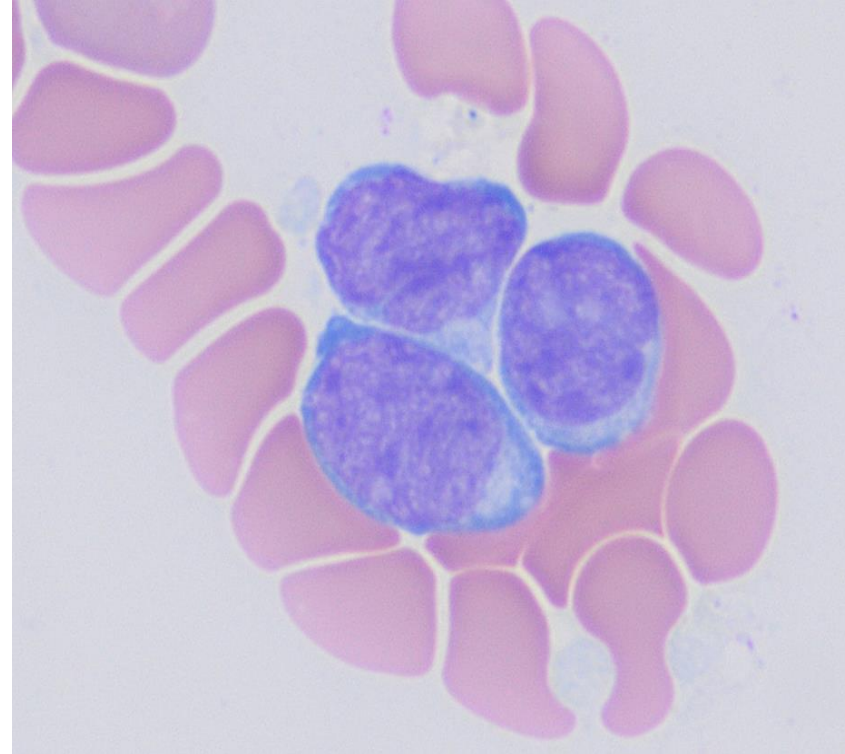

Case F

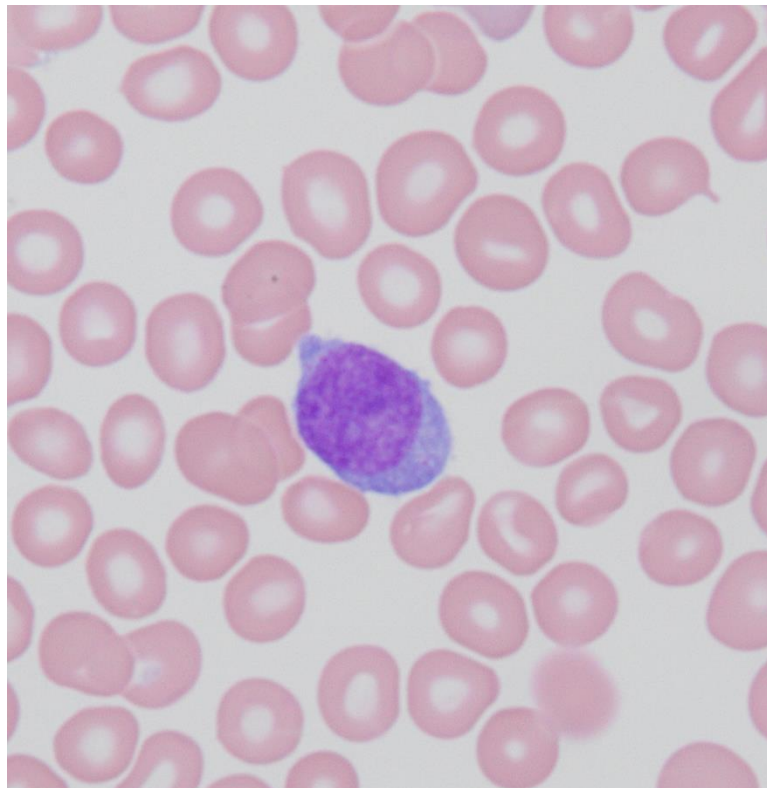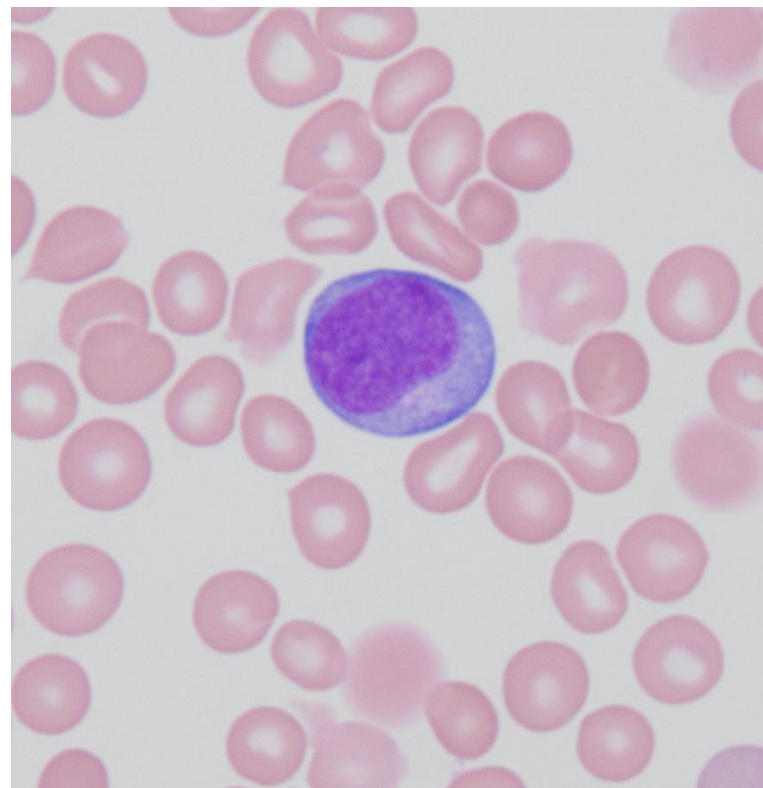

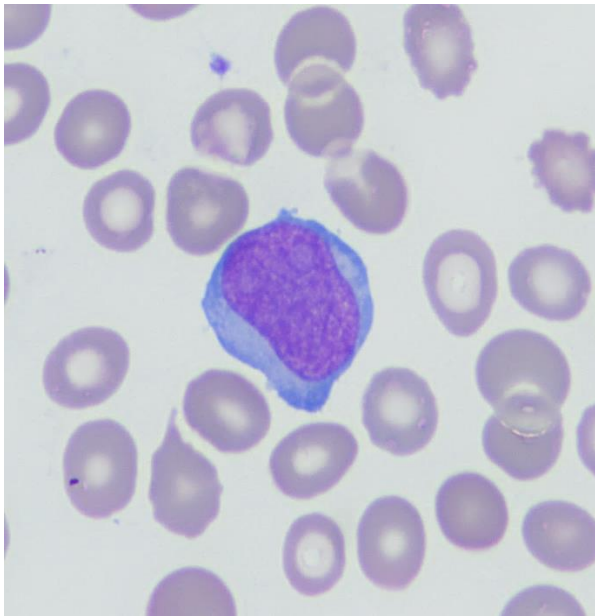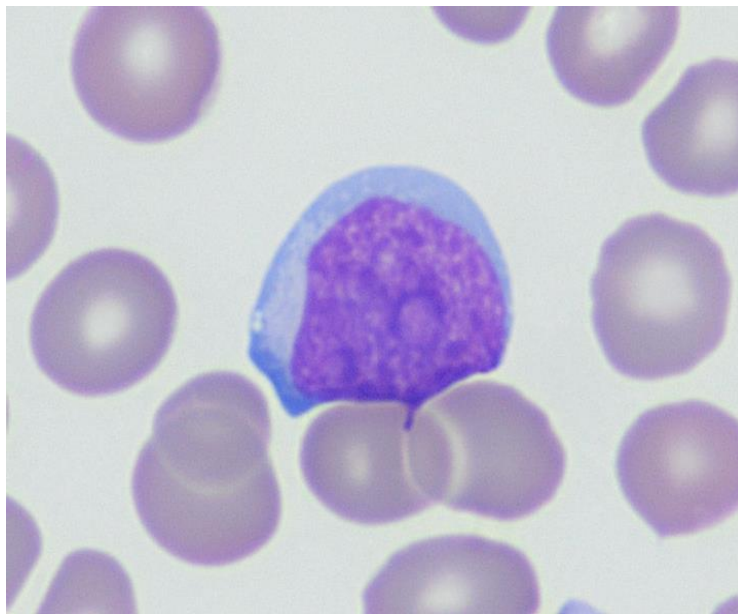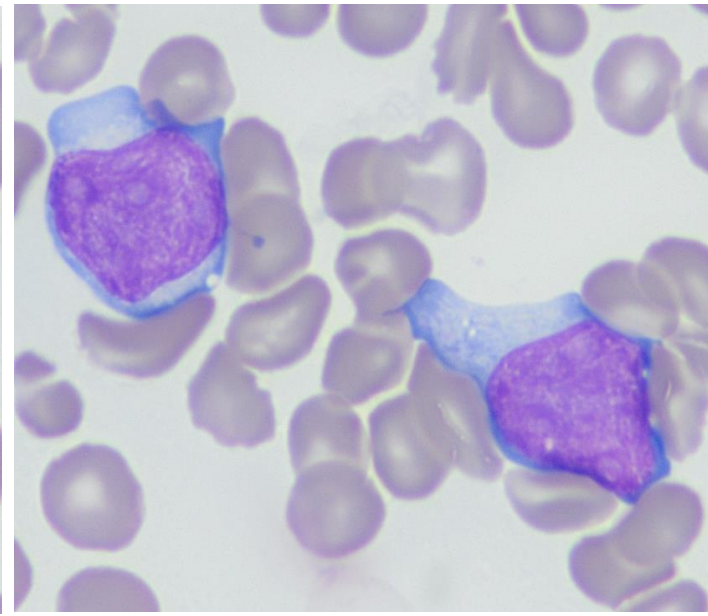

## Case H

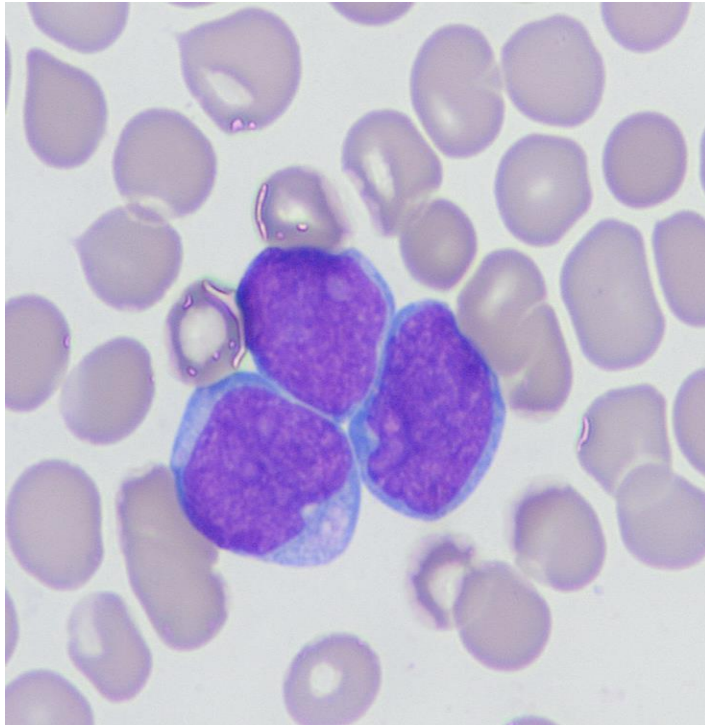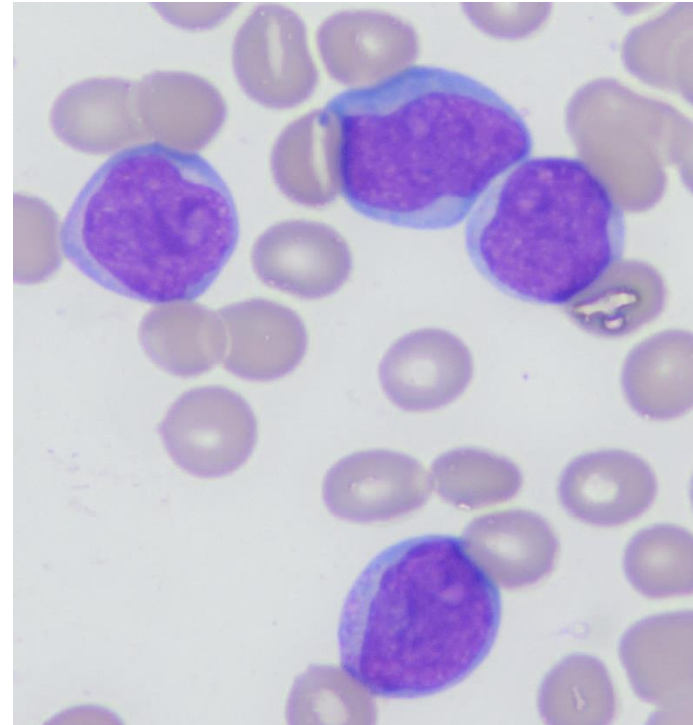

## Case I

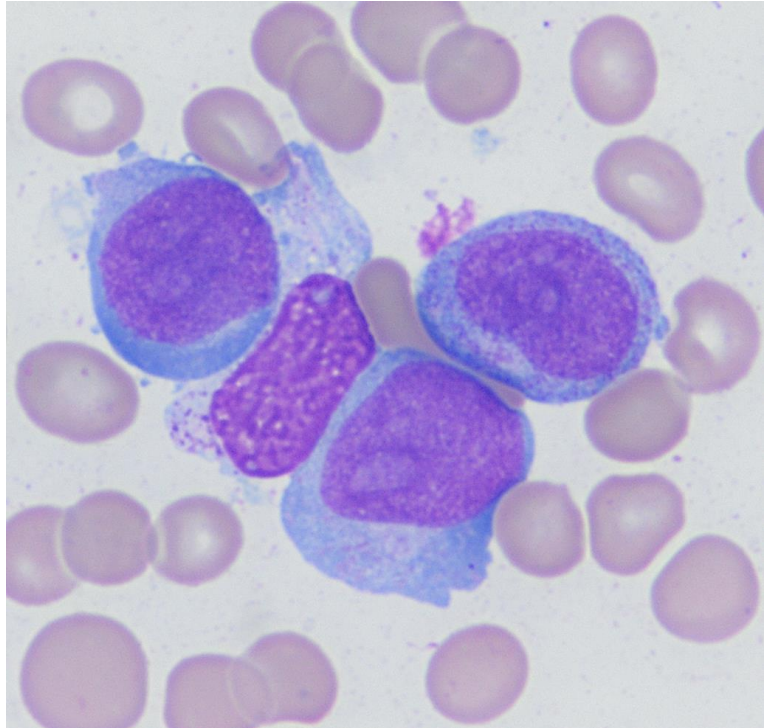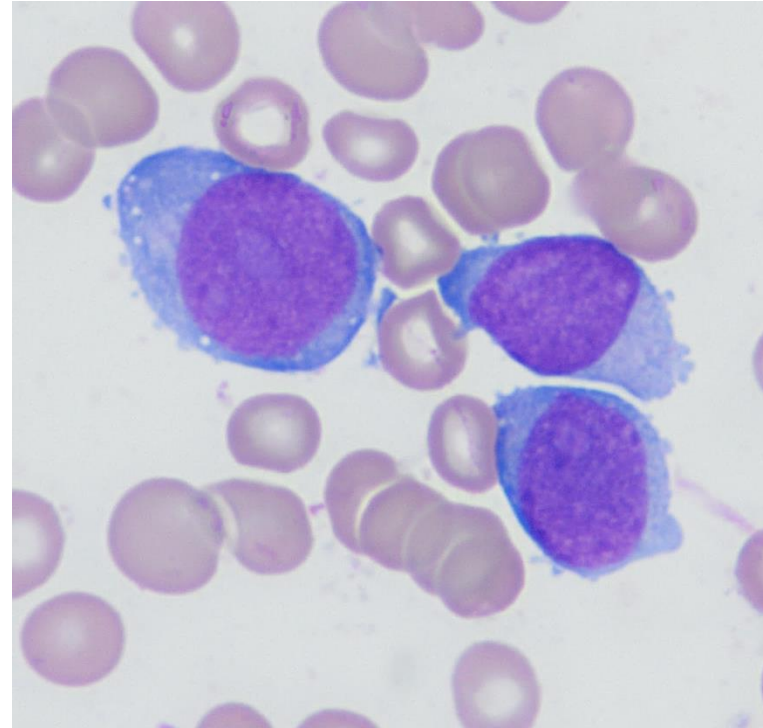

Case J

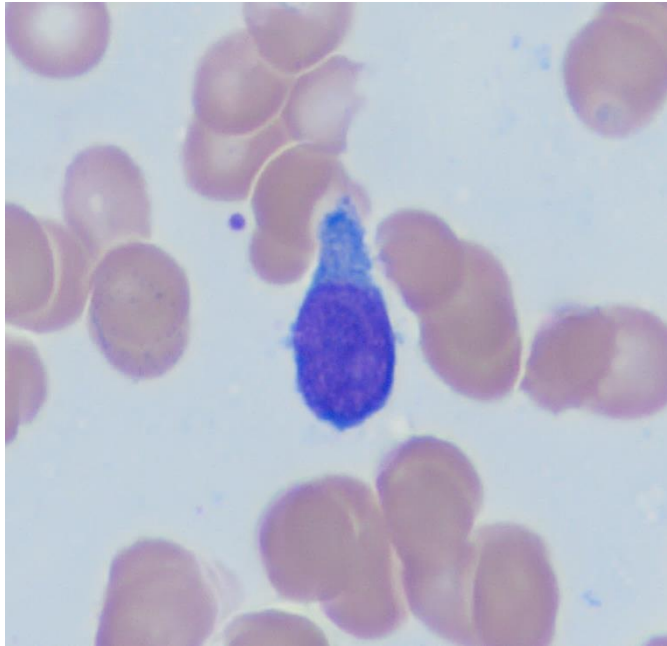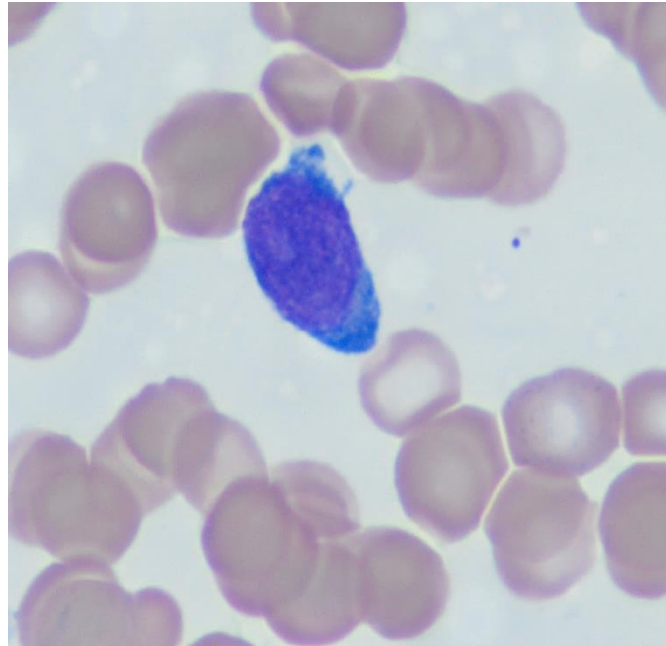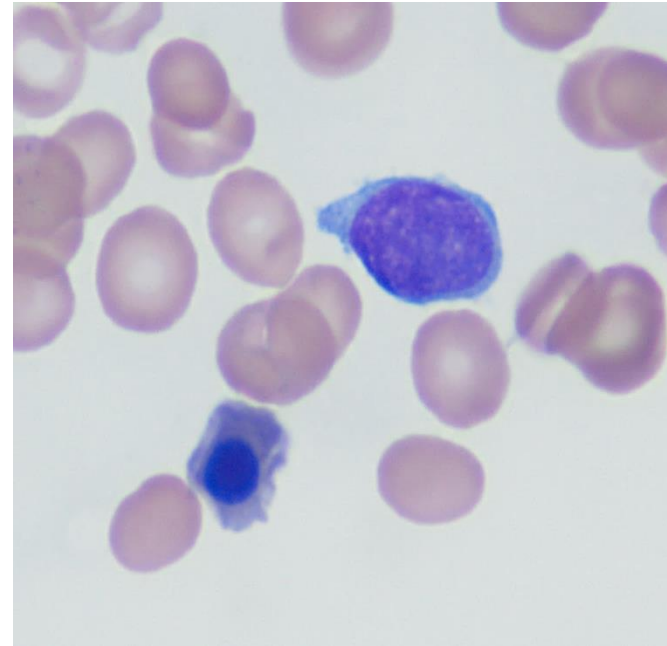

Case K

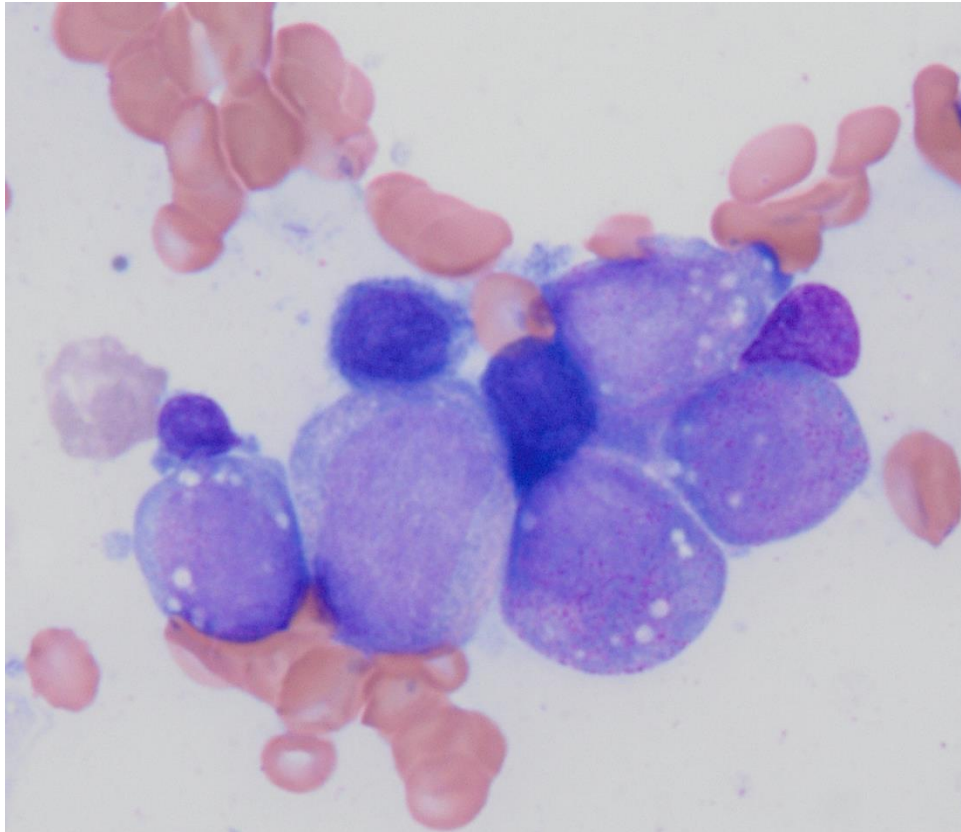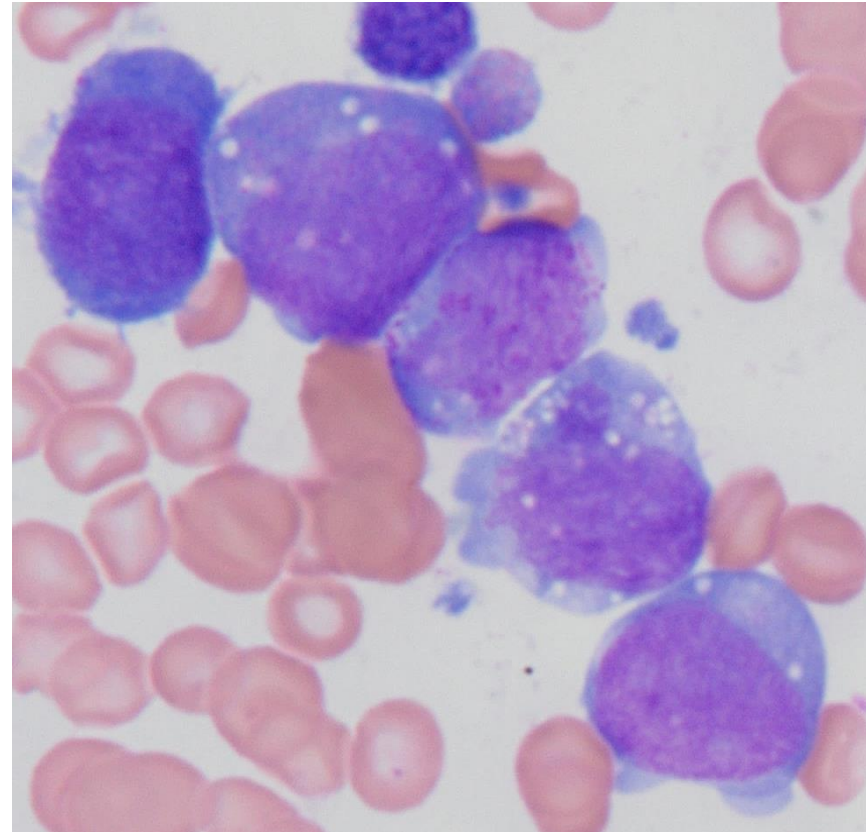

## Case L

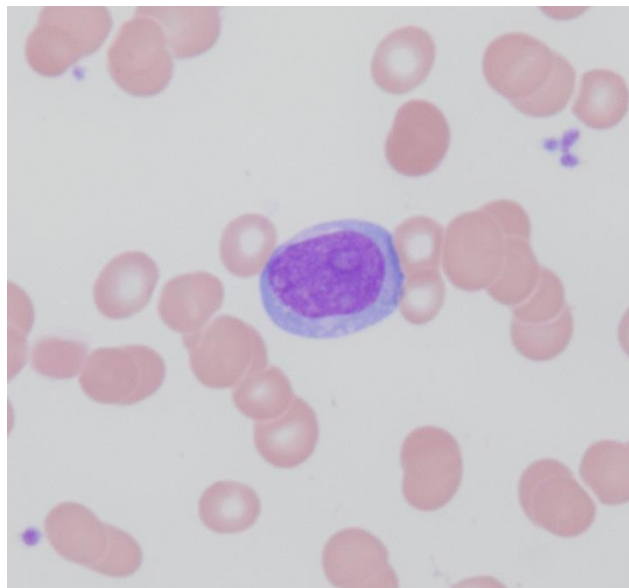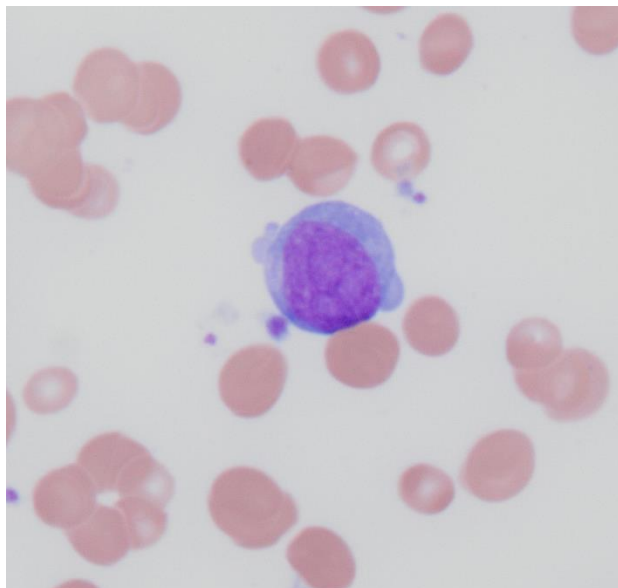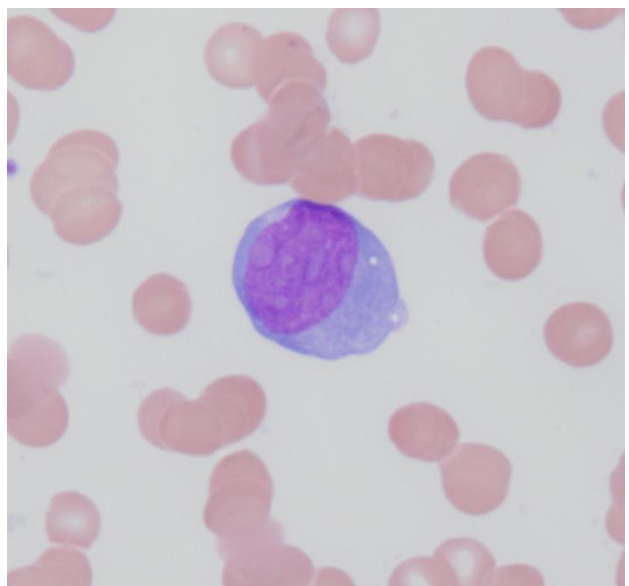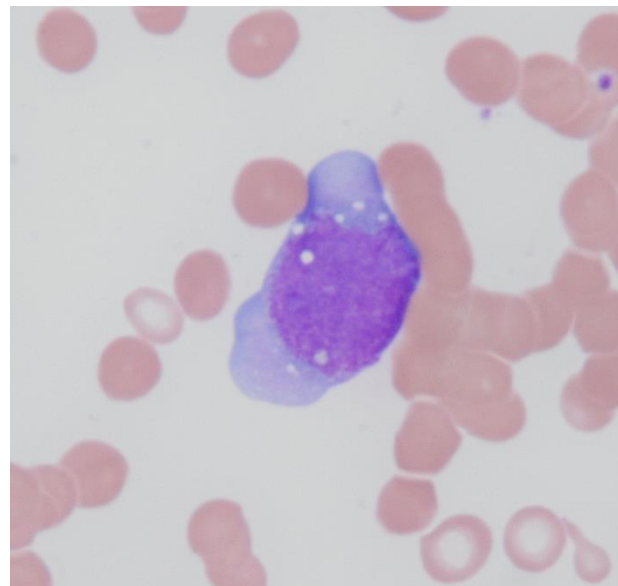

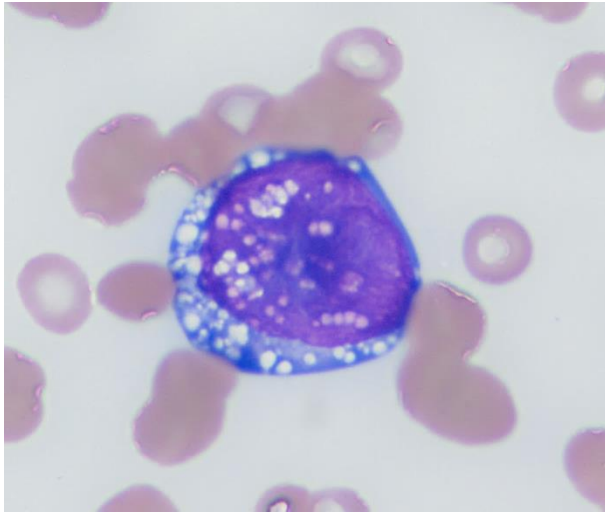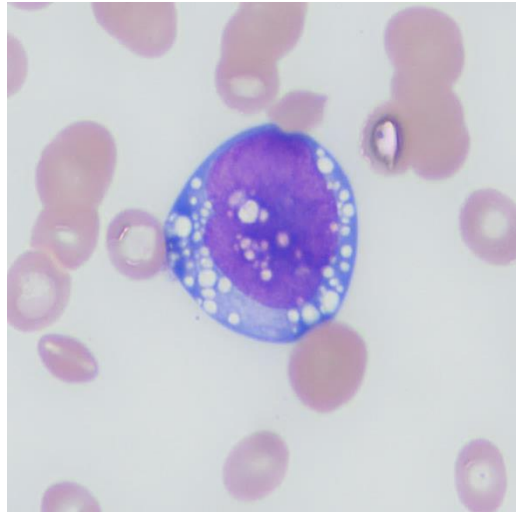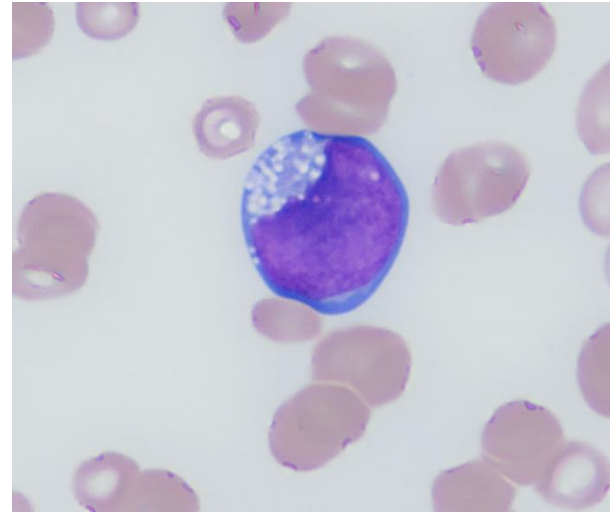

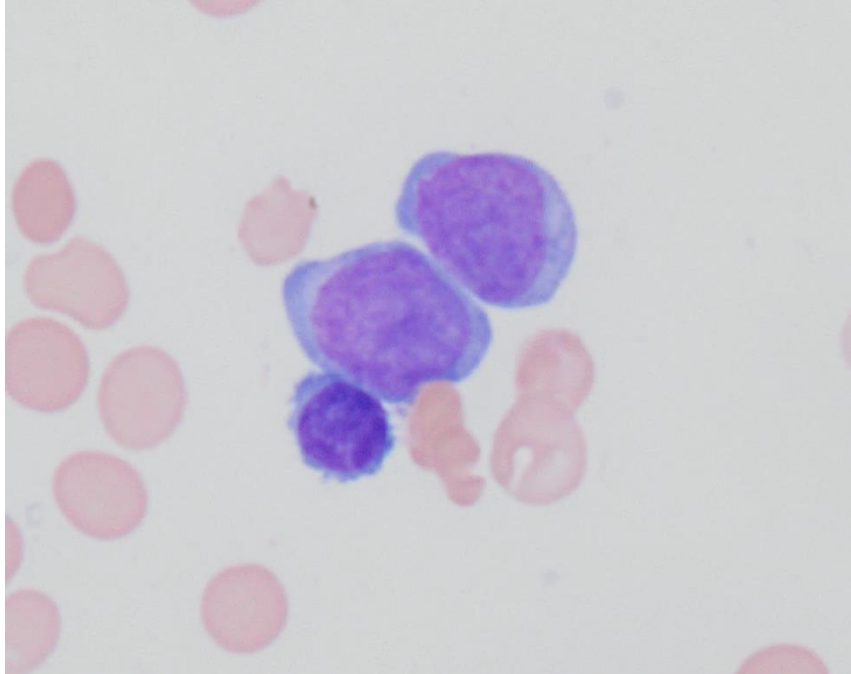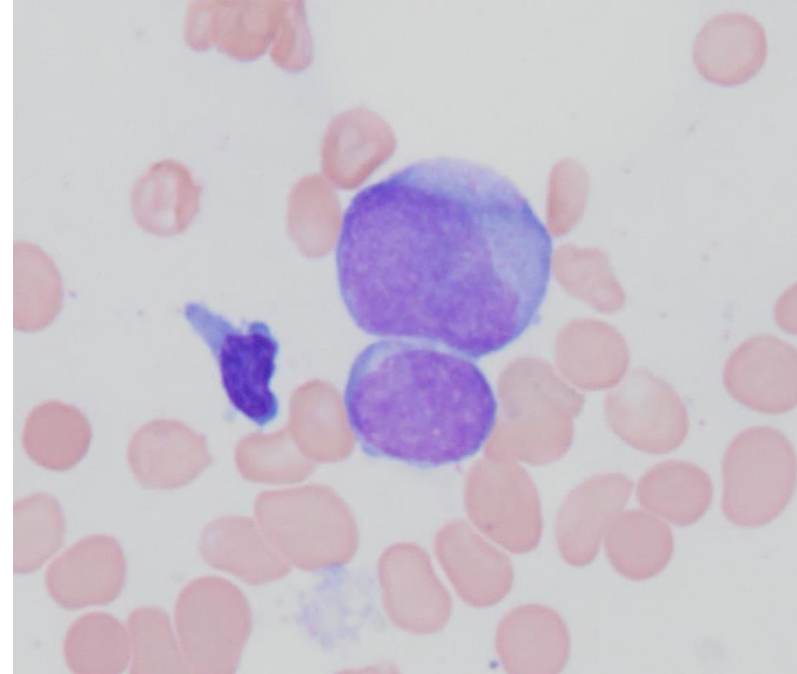

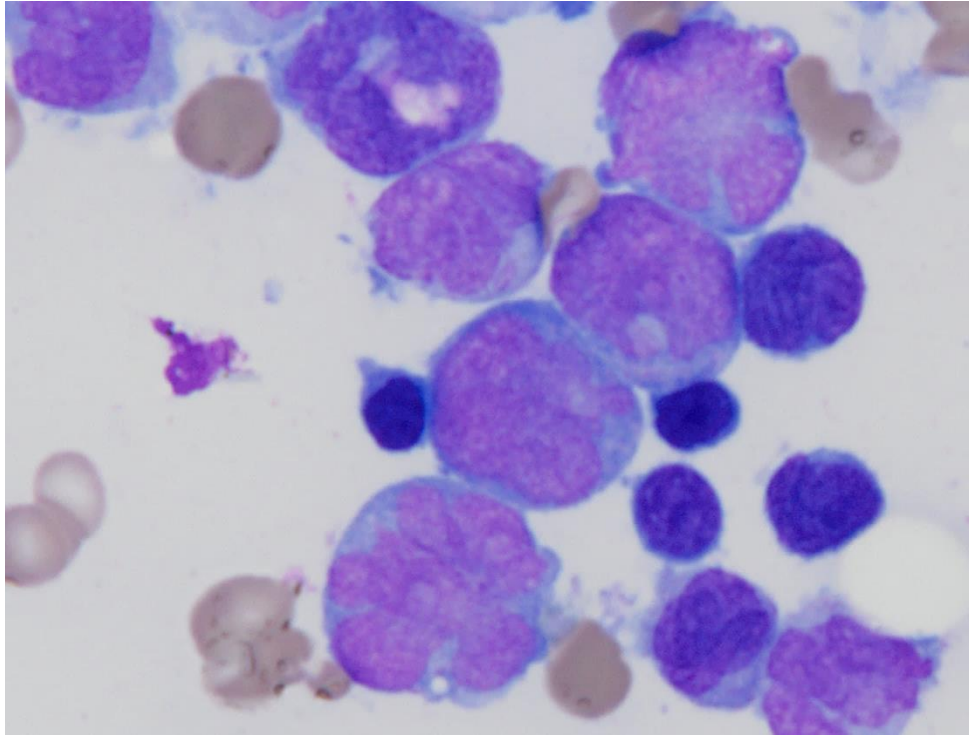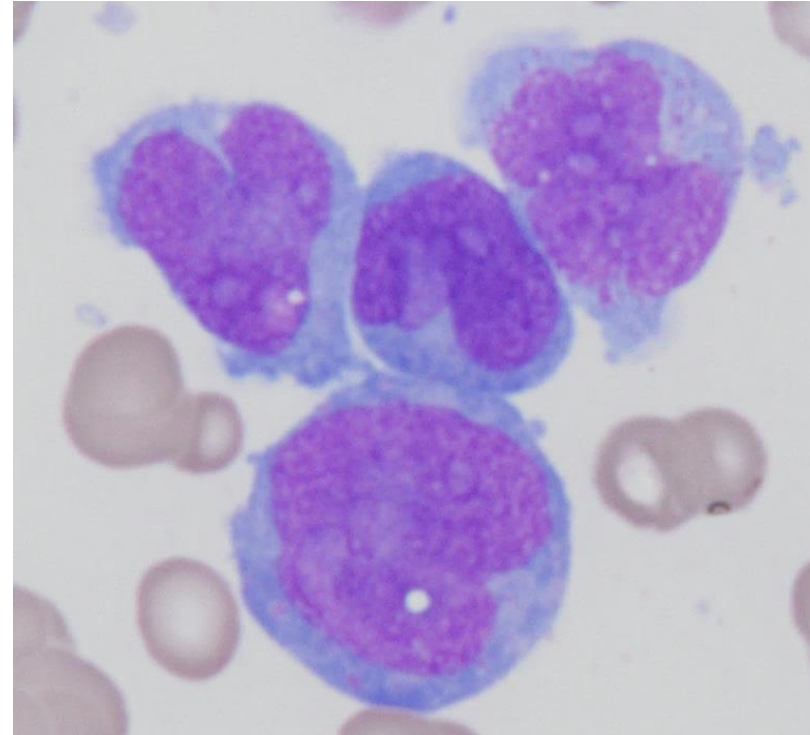

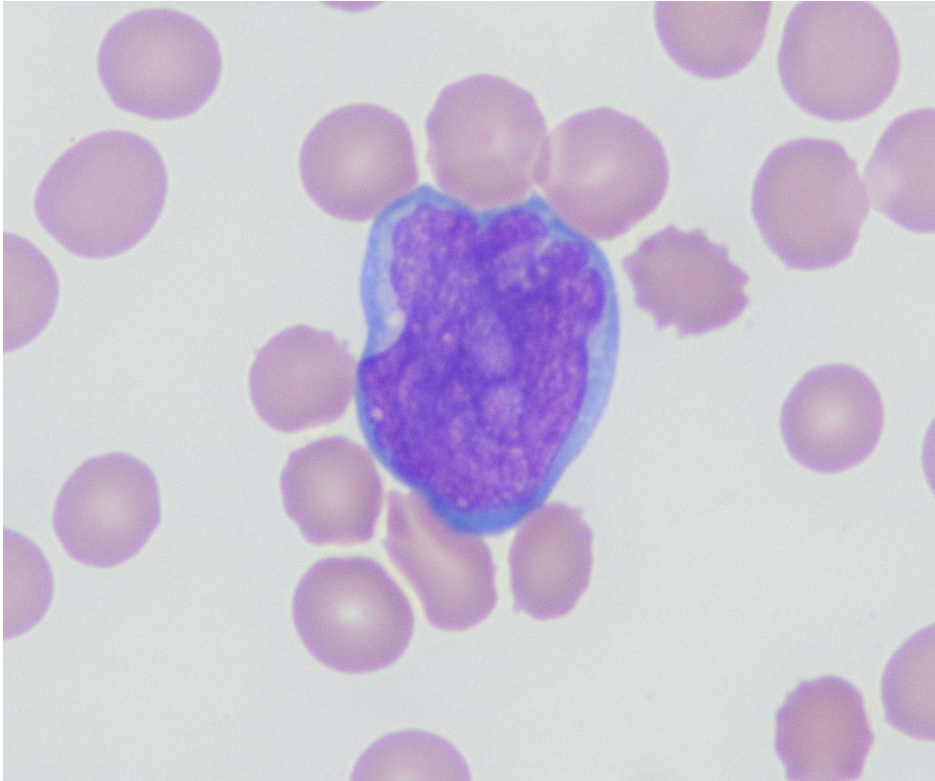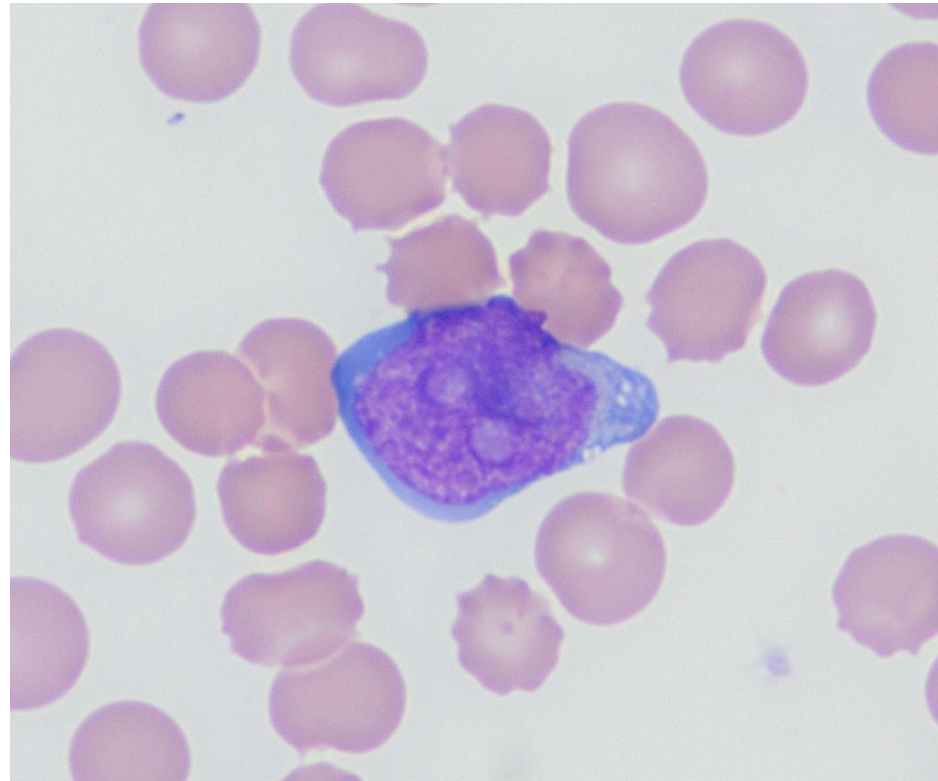

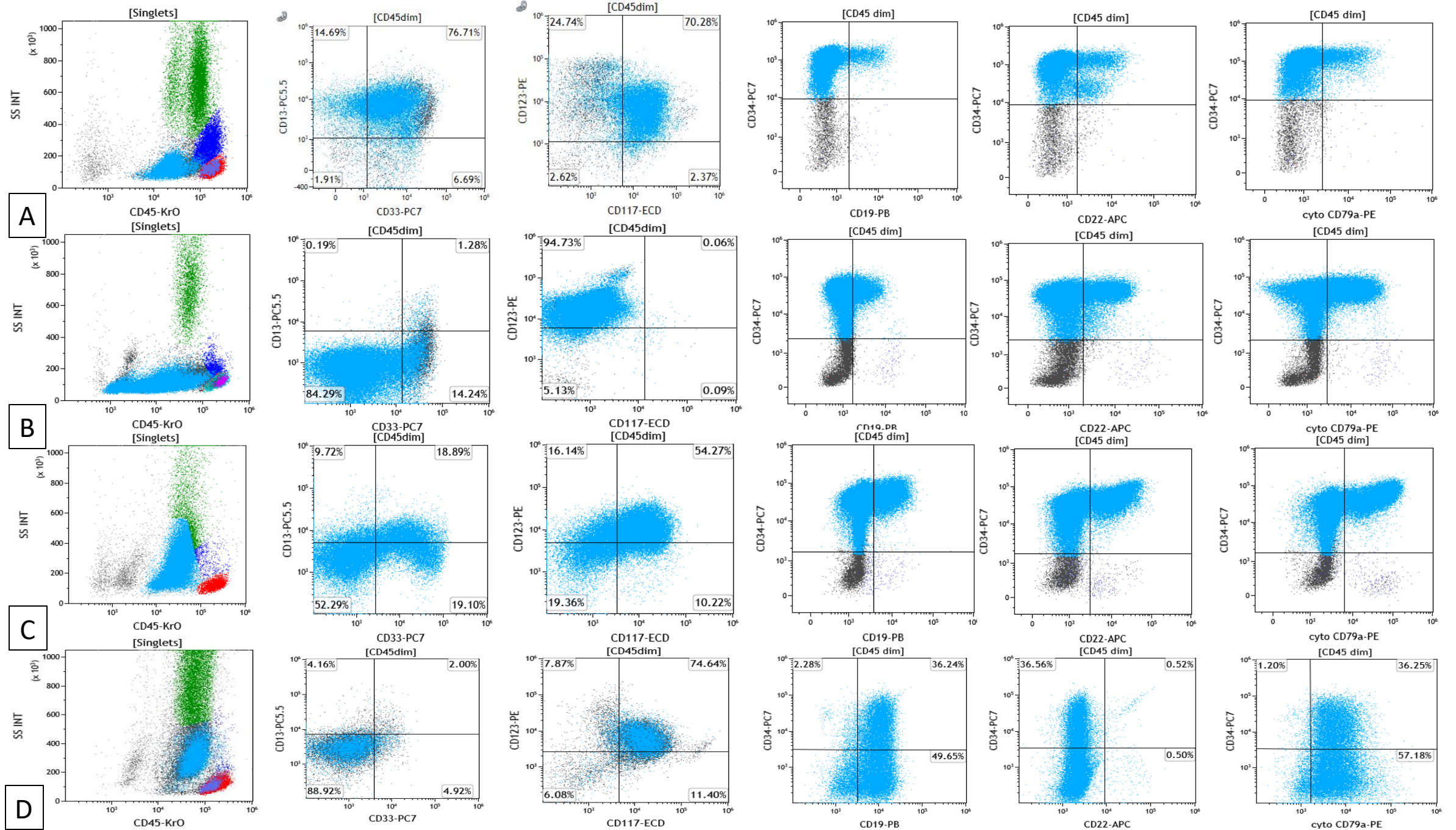

E

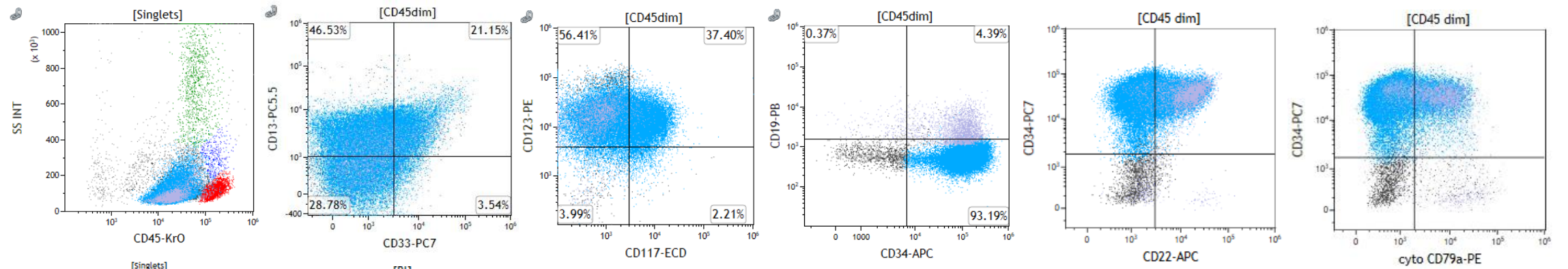

F

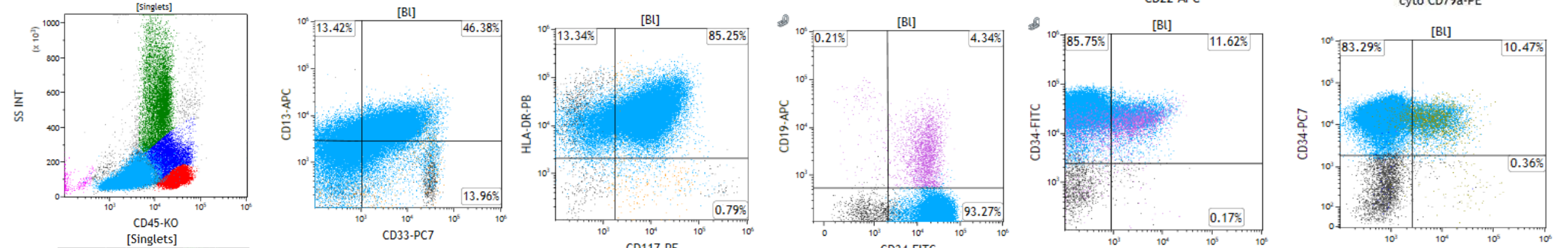

G

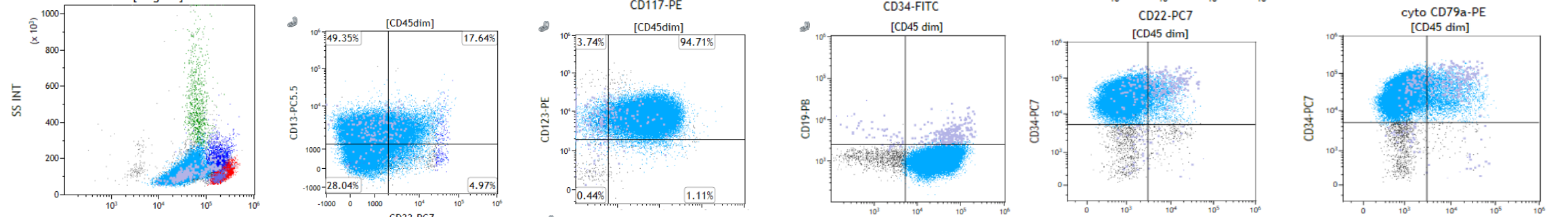

H

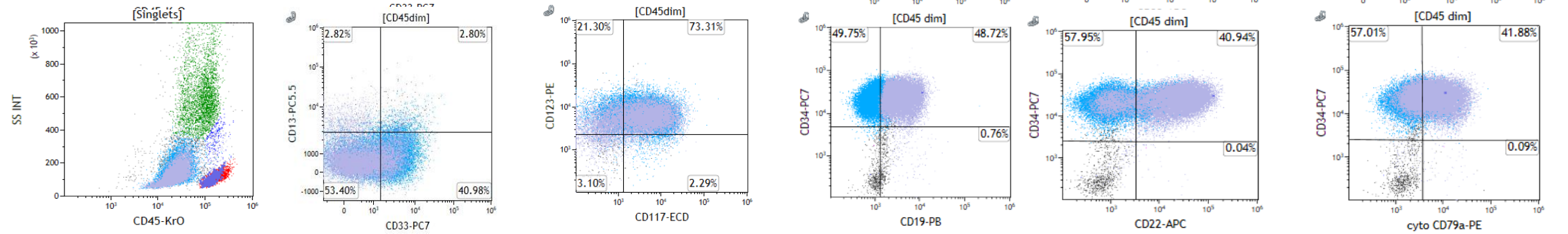

I

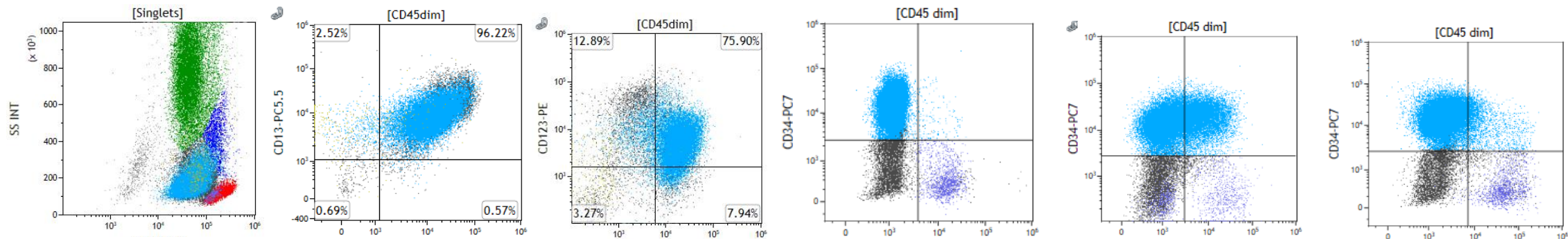

J

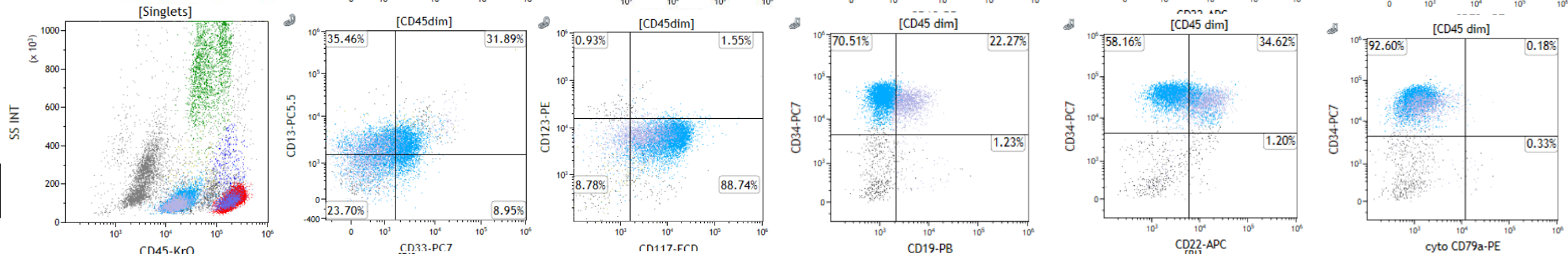

K

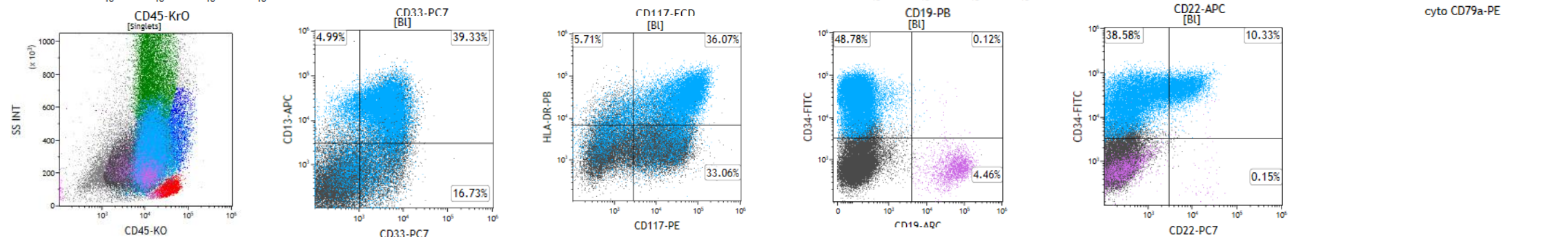

L

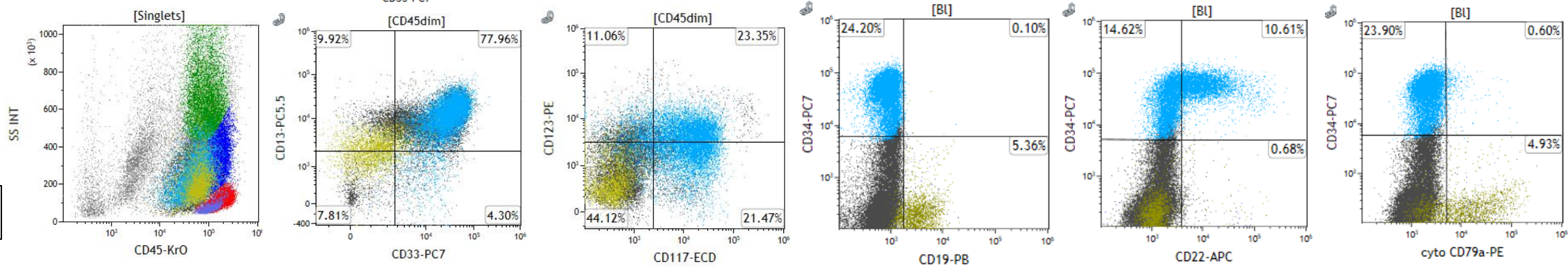

M

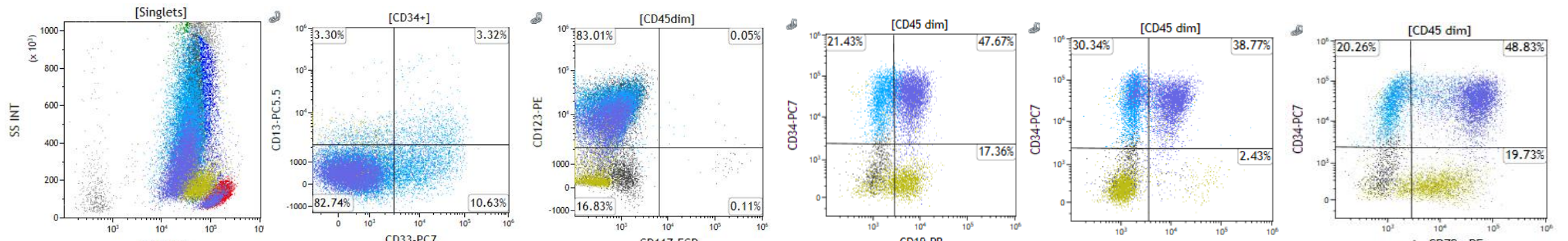

N

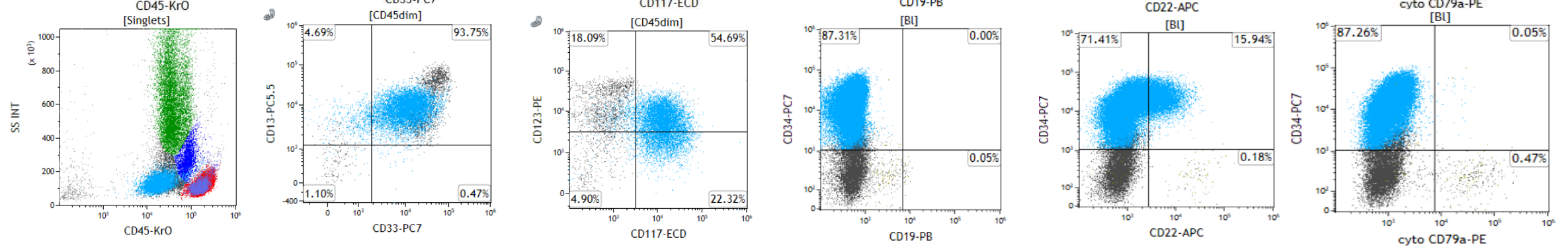

O

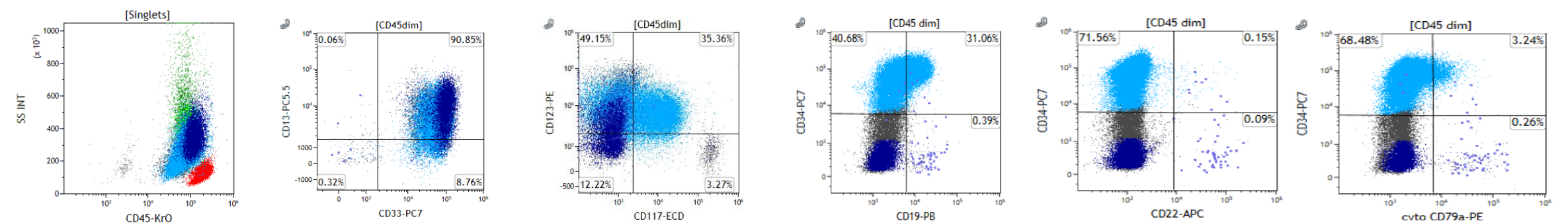

P

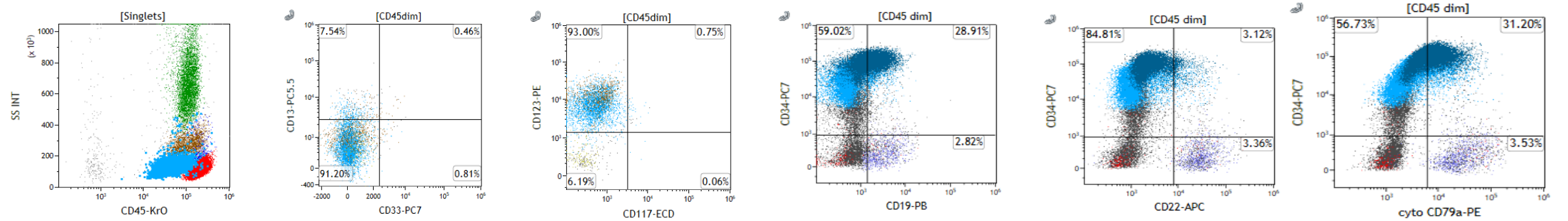

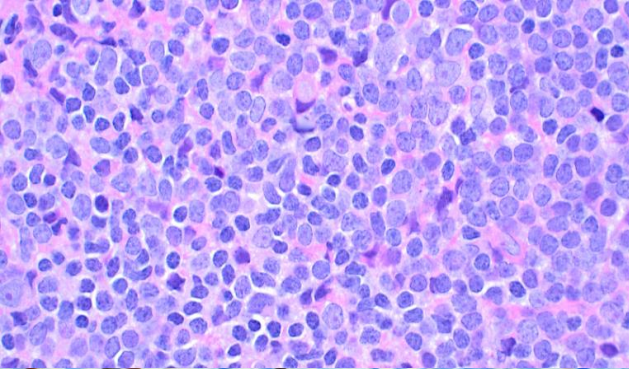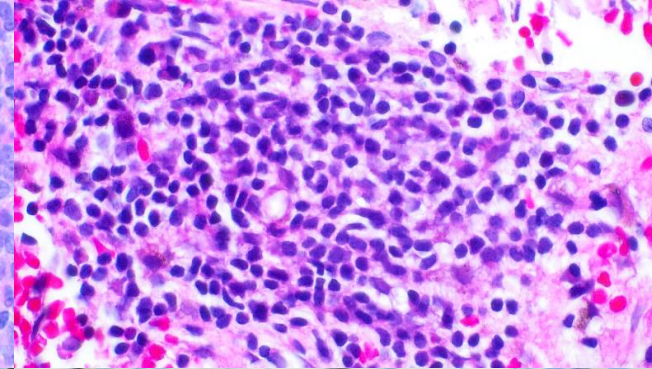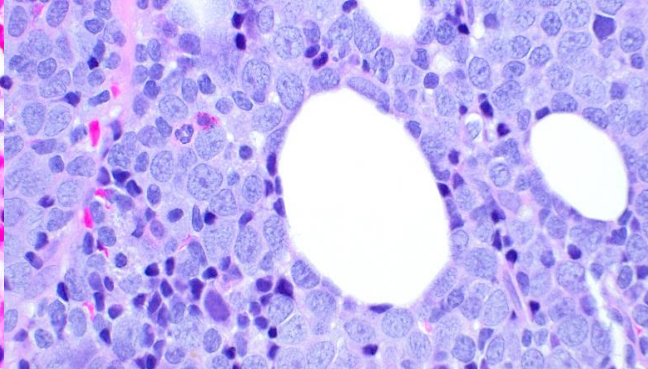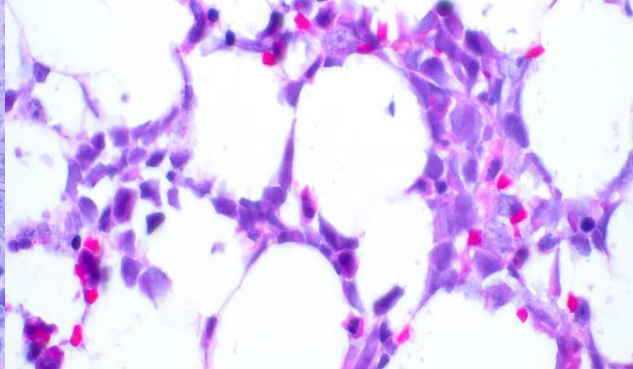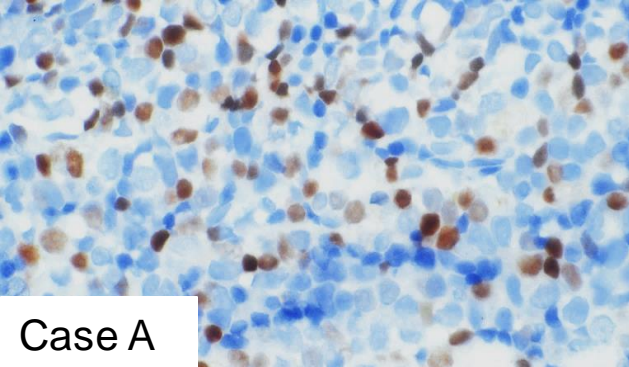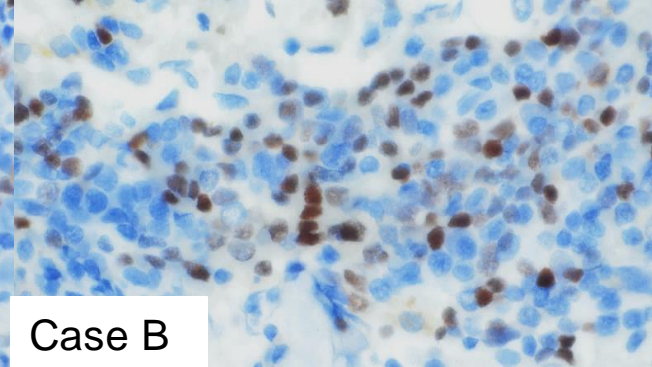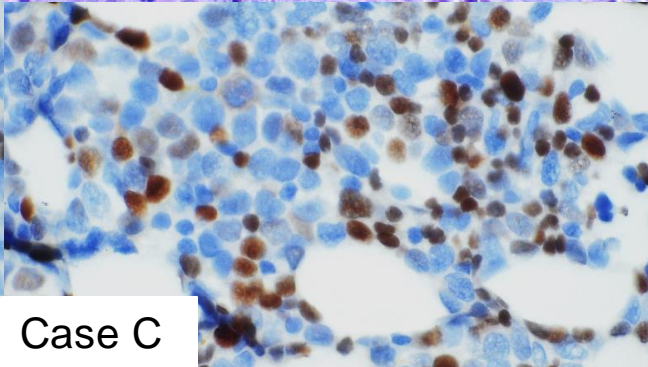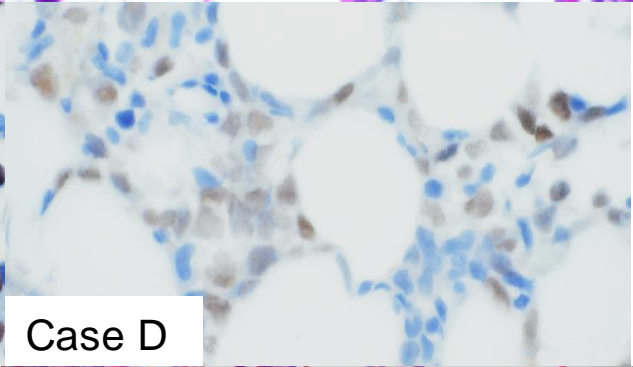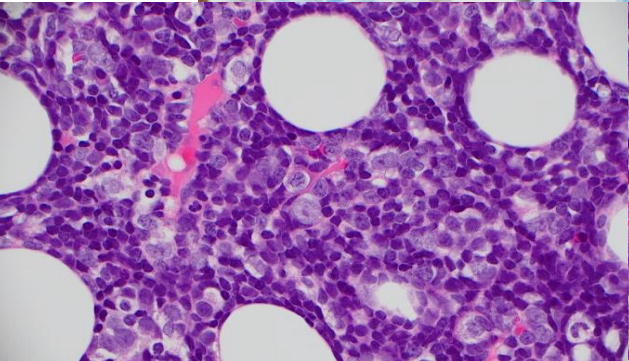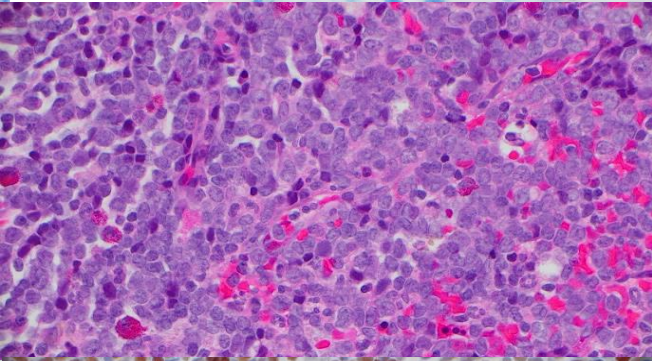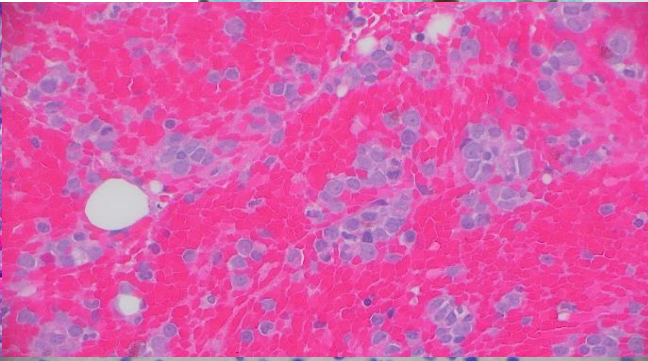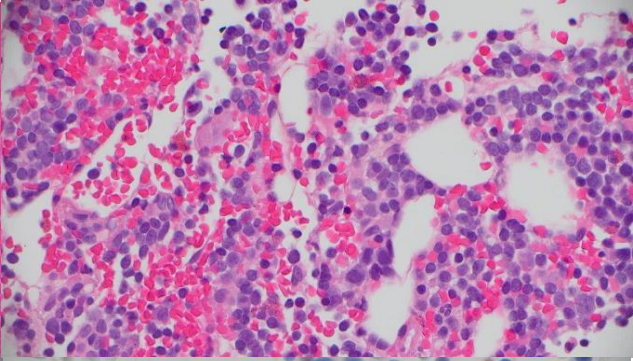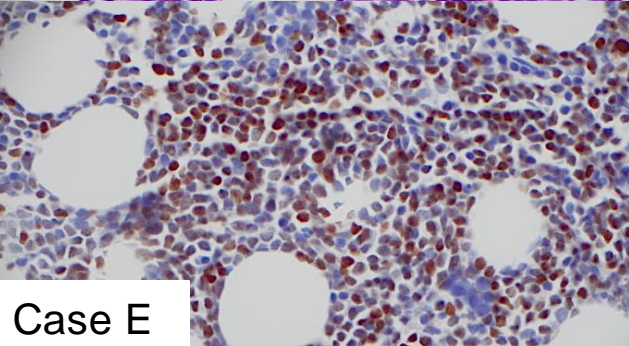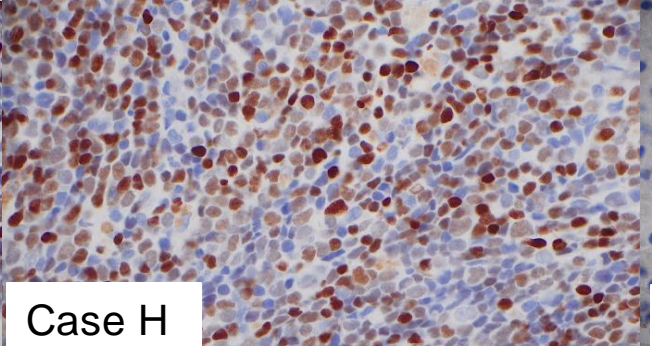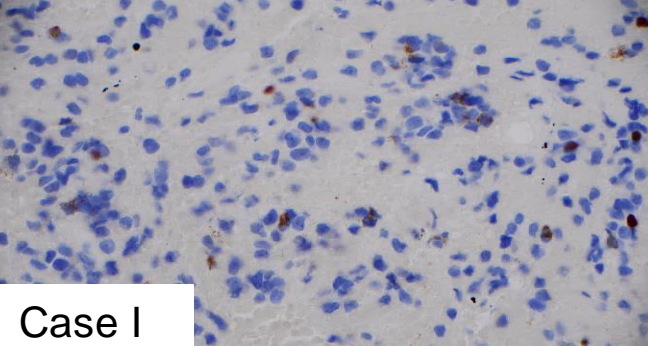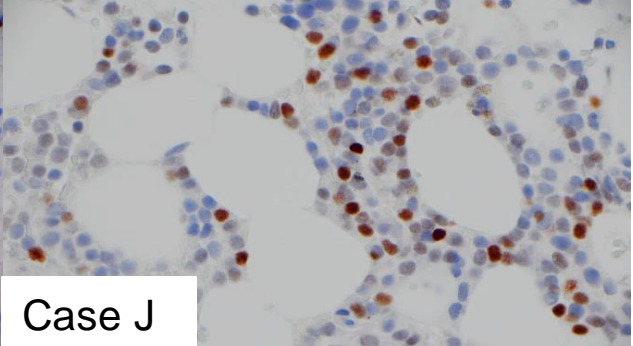

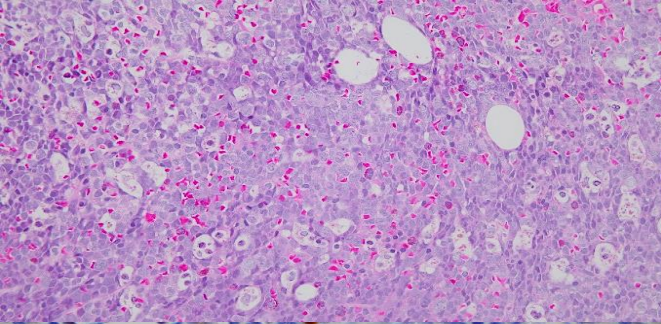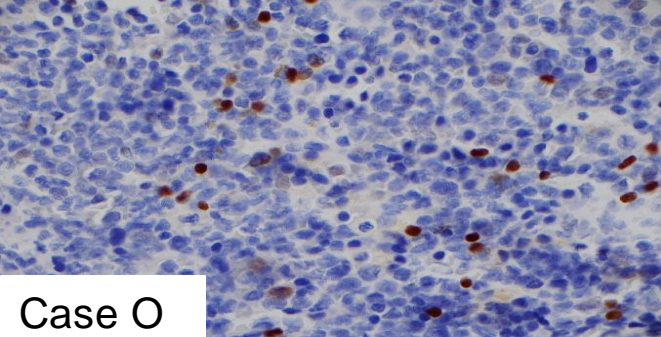

Case O

Supplement: Supplementary file 1 [file cancers-17-01354-s001.zip › cancers-3541185-supplementary.pdf]
